# Analysis of Functions of VIP1 and Its Close Homologs in Osmosensory Responses of *Arabidopsis thaliana*

**DOI:** 10.1371/journal.pone.0103930

**Published:** 2014-08-05

**Authors:** Daisuke Tsugama, Shenkui Liu, Tetsuo Takano

**Affiliations:** 1 Asian Natural Environmental Science Center (ANESC), The University of Tokyo, Nishi-tokyo-shi, Tokyo, Japan; 2 Laboratory of Plant Molecular Genetics, Graduate School of Agricultural and Life Sciences, The University of Tokyo, Bunkyo-ku, Tokyo, Japan; 3 Alkali Soil Natural Environmental Science Center (ASNESC), Northeast Forestry University, Harbin, China; Huazhong University of Science & Technology (HUST), China

## Abstract

VIP1 is a bZIP protein in *Arabidopsis thaliana*. VIP1 accumulates in the nucleus under hypo-osmotic conditions and interacts with the promoters of hypo-osmolarity-responsive genes, *CYP707A1* and *CYP707A3* (*CYP707A1/3*), but neither overexpression of VIP1 nor truncation of its DNA-binding region affects the expression of *CYP707A3* in vivo, raising the possibility that VIP and other proteins are functionally redundant. Here we show further analyses on VIP1 and its close homologs, namely, Arabidopsis group I bZIP proteins. The patterns of the signals of the GFP-fused group I bZIP proteins were similar in onion and Arabidopsis cells, suggesting that they have similar subcellular localization. In a yeast one-hybrid assay, the group I bZIP proteins caused reporter gene activation in the yeast reporter strain. VIP1 and other group I bZIP proteins showed positive results in a yeast two-hybrid assay and a bimolecular fluorescence complementation assay, suggesting that they physically interact. These results support the idea that they have somewhat similar functions. By gel shift assays, VIP1-binding sequences in the *CYP707A1/3* promoters were confirmed to be AGCTGT/G. Their presence in the promoters of the genes that respond to hypo-osmotic conditions was evaluated using previously published microarray data. Interestingly, a significantly higher proportion of the promoters of the genes that were up-regulated by rehydration treatment and/or submergence treatment (treatment by a hypotonic solution) and a significantly lower proportion of the promoters of the genes that were down-regulated by such treatment shared AGCTGT/G. To further assess the physiological role of VIP1, constitutively nuclear-localized variants of VIP1 were generated. When overexpressed in Arabidopsis, some of them as well as VIP1 caused growth retardation under a mannitol-stressed condition, where VIP1 is localized mainly in the cytoplasm. This raises the possibility that the expression of VIP1 itself rather than its nuclear localization is responsible for regulating the mannitol responses.

## Introduction

Water availability is a key factor determining the growth, productivity, and distribution of plants. When plants are dehydrated or rehydrated, expression of certain subsets of genes is changed, and this contributes to plant adaptation to dehydration or rehydration. For example, in *Arabidopsis thaliana*, dehydration induces the expression of *NCED3*, which plays a role in biosynthesis of a stress-related phytohormone, abscisic acid (ABA). This enhances the ABA level and thereby increases the stress tolerance of plants [1]–[4]. On the other hand, rehydration induces the expression of *CYP707A1* and *CYP707A3* (*CYP707A1/3*). CYP707A1/3 catalyze 8′-hydroxylation of ABA to decrease the ABA level [3]–[5].

Dehydration- and/or rehydration-responsive genes are thought to be under the control of osmosensory signaling, but upstream components of the plant osmosensory signaling are largely unknown. A transmembrane histidine kinase, Sln1, is thought to be an osmosensor in baker's yeast (*Saccharomyces cerevisiae*) ([6], for a review), and one of the Arabidopsis histidine kinases, ATHK1, has been suggested to be involved in dehydration responses. Knockout of *ATHK1* decreases drought tolerance and expression levels of ABA-responsive genes, while overexpression of *ATHK1* increases drought tolerance and the expression levels of the ABA-responsive genes [7]–[10]. However, it is still unclear how ATHK1 mediates transduction of dehydration signal.

VIP1 (VirE2-interacting protein 1) is an Arabidopsis bZIP protein that has roles in *Agrobacterium* responses [11]–[13], pathogen responses [14], [15] and low-sulfur responses [16]. In additions to these responses, we recently showed that VIP1 is involved in the Arabidopsis osmosensory responses. VIP1 is localized mainly in the cytoplasm both when extracellular osmolarity is stable and when cells are exposed to hyper-osmotic conditions. When cells are exposed to hypo-osmotic conditions, VIP1 accumulates in the nucleus and interacts with *CYP707A1/3* promoters. On the other hand, neither overexpression of VIP1 nor truncation of its C-terminal DNA-binding region affects the expression of *CYP707A3* in vivo [17], raising the possibility that VIP1 and other proteins redundantly regulate the expression hypo-osmolarity-responsive genes such as *CYP707A3*. Homologs of VIP1 are thought to be candidates for such factors. Arabidopsis bZIP proteins have been classified into 11 groups on the basis of similarities and features of their amino acid sequences. This classification somewhat reflects the functions of plant bZIP proteins. For example, group A bZIP proteins redundantly function in ABA signaling ([18], for a review). VIP1 belongs to Arabidopsis group I bZIP protein. The Arabidopsis group I contains 11 other members, but they have not been well characterized. Here we show that VIP1 and other group I bZIP proteins are redundant in the subcellular localization and the transcriptional activation function, and can interact with each other. We also further address the relationship between the group I bZIP proteins and Arabidopsis osmosensory responses.

## Materials and Methods

### Plant materials and growth conditions

*Arabidopsis thaliana* ecotype Columbia-0 (Col-0) was used throughout the experiments. Seeds of *vip1* (SALK_001014C) [13] were obtained from ABRC (Arabidopsis Biological Resource Center, https://abrc.osu.edu/). The T-DNA insertion was confirmed by genomic PCR analysis as previously described [13]. Seeds were surface sterilized and sown on 0.8% agar containing 0.5× MS salts (Wako), 1% (w/v) sucrose and 0.5 g/l MES, pH 5.8, with 0, 200 or 250 mM mannitol or 0.25 µM ABA, chilled at 4°C in the dark for 48 h (stratified), and germinated at 22°C. Plants were grown at 22°C under 16-hour light/8-hour dark condition (light intensity 120 µmol·m^−2^·s^−1^). Arabidopsis transformation was performed by the *Agrobacterium*-mediated floral-dip method [19], and T2 generation plants were used for analyses. GFP fluorescence in individual plants was checked immediately before measuring cotyledon lengths to confirm the expression of the transgenes.

### Reverse transcription-PCR (RT-PCR)

To analyze stage- and tissue-specific expression of the group I bZIP protein genes, 10-day-old seedlings and roots, leaves, and inflorescences of mature wild-type plants were sampled. To analyze gene expression under a hypo-osmotic condition, 2-week-old wild-type seedlings were sampled 0, 15 and 90 minutes after being submerged in 20 mM Tris-HCl, pH 6.8. To analyze transgene expression, 10-day-old seedlings of each transgenic line were sampled. To analyze gene expression in transgenic plants, 18-day-old seedlings grown in the presence of 0 or 200 mM mannitol were sampled. In each condition, 10–20 plants were sampled and used for RNA extraction for one biological replicate. Total RNA was extracted as previously described [20] and cDNA was synthesized from 3 µg of the total RNA with PrimeScript Reverse Transcriptase (Takara Bio) using an oligo (dT) primer. Reaction mixtures were diluted 25 times with distilled water and used as templates for RT-PCR. To quantify the absolute transcript levels of the group I bZIP protein genes, cDNA clones were obtained from RIKEN BRC Experimental Plant Division [21] (see Table S1 for their clone names). Partial fragments of cDNA of *UNE4* (*AT2G12940*), *AtbZIP71* (*AT2G24340*), and *AtbZIP74* (*AT2G21235*) were amplified by RT-PCR using cDNA synthesized from total RNA from Arabidopsis seedlings as template, and cloned into pBluescript SK^−^. Partial fragments of cDNA clones of *AtbZIP31* (*AT2G13150*) and *AtbZIP33* (*AT2G12900*), were obtained by PCR as described in Table S2. These plasmids and PCR products were used to make standard curves.

GoTaq Green Master Mix (Promega) was used for semi-quantitative RT-PCR, and GoTaq qPCR Master Mix (Promega) for quantitative RT-PCR. Quantitative PCR was run using a StepOne Real-Time PCR System (Applied Biosystems). The comparative C_T_ method was used to calculate the relative expression levels, and the standard curve method was used to calculate the absolute transcript levels. *UBQ5* was used as an internal control gene for normalization. Primers used for RT-PCR are listed in Table S2 (for the group I bZIP protein genes), Table S3 (for the transgenes) and Dataset S2 (for the genes that have AGCTGT/G in their promoters).

### Yeast one-hybrid (Y1H) and two-hybrid (Y2H) assays

For Y1H and Y2H, the open reading frames (ORFs) of the group I bZIP protein genes and truncated versions of *VIP1* were amplified by PCR using the RIKEN cDNA clones as template, and cloned into pGADT7-Rec and pGBKT7 (Clontech). Primers and restriction sites used to generate the constructs are listed in Table S4. For Y1H, the *Saccharomyces cerevisiae* strain AH109 (Clontech) was transformed with the pGBK constructs. For Y2H, AH109 was co-transformed with pGBKT7 containing the ORF of the C-terminal region (amino acids 165–341) of VIP1 (pGBK-VIP1ΔN164) and the pGAD constructs. Reporter gene activation was checked by observing cell growth on SD (synthetic dextrose) media lacking adenine and histidine as previously described [17], [22].

### Bimolecular fluorescence complementation (BiFC) and GFP localization studies

The ORFs of the group I bZIP protein genes and truncated versions of *VIP1* were amplified by PCR using the RIKEN cDNA clones as template, and inserted into pBS-35S-nYFP-2, pBS-35SMCS-cYFP [17], pBS-35SMCS-GFP [23] and/or pBI121-35SMCS-GFP [24]. Primers and restrictions sites used to generate the constructs are listed in Table S5. The ORFs of VIP1 variants that have point-mutations in the putative phosphorylation sites and the ORF of a VIP1 variant that has the SV40 NLS in its N-terminus were generated and inserted into pBS-35SMCS-GFP and/or pBI121-35SMCS-GFP as described in Table S6. The ORFs of the VIP1 variants in the generated constructs were sequenced to confirm the truncations, the mutations and the fusion of the SV40 NLS. The BiFC constructs (nYFP and cYFP constructs) and the pBS-35SMCS-GFP constructs were used for transient expression experiments using onion cells, while the pBI121-35SMCS-GFP constructs were used for Arabidopsis transformation. Onion epidermal cells were transformed by particle bombardment as previously described [25]. Arabidopsis transformation was performed as described above. Fluorescence was observed using an epifluorescence microscope (BX51, Olympus) in all the experiments.

### Gel shift assay

To express the GST-fused C-terminal region (the amino acid position 292–553) of AtbZIP29 (GST-AtbZIP29ΔN291), the pGBK construct containing *AtbZIP29* (see above and Table S4) was digested by *Eco*RI and *Sal*I, and the resultant fragment corresponding to *AtbZIP29ΔN291* was inserted into the *Eco*RI-*Sal*I site of pGEX-6P-3 (GE Healthcare). The resultant construct was transformed into the *Escherichia coli* strain BL21 (DE3). To induce GST-AtbZIP29ΔN291, the transformed *E. coli* cells were grown in liquid LB medium at 37°C. When the OD_600_ reached 0.5, IPTG was added to the medium to a final concentration of 0.2 mM. The cells were further grown at 28°C for 3 h, collected by centrifugation (12000×g, 1 min), resuspended in 1× TBS (150 mM NaCl, 20 mM Tris-HCl, pH 7.5) containing 1 mg/ml lysozyme, frozen at −80°C, and thawed at room temperature. The freezing and thawing were repeated twice more, and 2 units recombinant DNase I was added to the solution. The solution was incubated for 20 min at room temperature, and centrifuged at 12000×g for 5 min at room temperature. GST-AtbZIP29ΔN291 in the supernatant was bound to Glutathione Sepharose 4 Fast Flow (GE Healthcare), washed five times by 1× TBS, eluted in 20 mM reduced glutathione in 50 mM Tris-HCl, pH 8.0, as manufacturer's instructions, and used as the purified protein sample.

Gel shift assays were performed using DIG-labeled DNA probes and the purified protein samples as previously described [17]. The sequences of the probes are provided in Fig. S3. To prepare the competitors for Fig. S4 and Fig. S5, two complementary oligonucleotides were mixed in equal concentrations, incubated at 90°C for 5 min, and gradually cooled to room temperature. The sequences of the competitors are provided in Fig. S5. The competitors were used at 0.5 µM unless otherwise stated.

### Analysis of the VIP1-binding element in the promoters of the rehydration- and submergence-responsive genes

The lists of the rehydration- and submergence-responsive genes were obtained from the previous reports [26]–[29] (see Table 1 and Dataset S1 for the criteria of those genes). The list of the genes with promoters (regions either 500-bp or 1000-bp upstream of the start codon) containing the VIP1-binding element (AGCTGT or AGCTGG) was obtained with Patmatch in TAIR (https://www.arabidopsis.org/cgi-bin/patmatch/nph-patmatch.pl). These lists were used for one-sided binomial tests to evaluate the abundance of the VIP1-binding element in the promoters of the rehydration- and submergence-responsive genes. For the binomial tests, the numbers of the rehydration- and submergence-responsive genes were used as “the number of trials”, and the numbers of the rehydration- and submergence-responsive genes with promoters containing the VIP1-binding element were used as “the numbers of success”. For “the probabilities of success”, the total number of the genes with promoters containing the VIP1-binding element was divided by the total number of genes in the Arabidopsis genome, and the resultant values was used. Those numbers used for the binomial tests are shown in Table 1 and Dataset S1.

**Table 1:** Proportions of the genes with promoters (500 bp upstream of the start codon) containing AGCTGT/G in the submergence- and/or rehydration-responsive gene sets detected in the previously performed microarray analyses.

* ^1^Gene set	Proportion of genes with at least one AGCTGT/G	Cumulative binomial distribution	*4	Proportion of genes with at least two AGCTGT/G	Cumulative binomial distribution	*4
* ^1^Total in the genome	0.185	0.504		0.033	0.508	
* ^2^Up-regulated 30 min after submergence in root	0.263	0.986	+	0.068	0.983	+
* ^2^Up-regulated 30 min after submergence in shoot	0.194	0.652		0.032	0.724	
* ^2^Up-regulated 1 h after submergence in root	0.235	0.989	+	0.056	0.985	+
* ^2^Up-regulated 1 h after submergence in shoot	0.176	0.5		0.029	0.604	
* ^2^Up-regulated 3 h after submergence in root	0.198	0.839		0.069	∼1	++
* ^2^Up-regulated 3 h after submergence in shoot	0.147	0.103		0.021	0.239	
* ^2^Up-regulated 6 h after submergence in root	0.194	0.792		0.034	0.58	
* ^2^Up-regulated 6 h after submergence in shoot	0.166	0.159		0.028	0.32	
* ^2^Up-regulated 12 h after submergence in root	0.196	0.849		0.037	0.776	
* ^2^Up-regulated 12 h after submergence in shoot	0.175	0.244		0.028	0.247	
* ^3^Up-regulated by 2-h rehydration and 2-h dehydration	0.293	0.999	++	0.102	∼1	++
* ^2^Down-regulated 30 min after submergence in root	0.23	0.908		0.027	0.478	
* ^2^Down-regulated 30 min after submergence in shoot	0.143	0.618		0	0.789	
* ^2^Down-regulated 1 h after submergence in root	0.193	0.683		0.017	0.037	*
* ^2^Down-regulated 1 h after submergence in shoot	0.125	0.409		0	0.582	
* ^2^Down-regulated 3 h after submergence in root	0.187	0.561		0.036	0.729	
* ^2^Down-regulated 3 h after submergence in shoot	0.133	0.11		0.031	0.587	
* ^2^Down-regulated 6 h after submergence in root	0.185	0.498		0.036	0.736	
* ^2^Down-regulated 6 h after submergence in shoot	0.142	0.01	*	0.033	0.569	
* ^2^Down-regulated 12 h after submergence in root	0.18	0.304		0.033	0.529	
* ^2^Down-regulated 12 h after submergence in shoot	0.155	0.008	**	0.026	0.106	
* ^3^Down-regulated by 2-h rehydration after 2-h dehydration	0.16	0.171		0.016	0.0786	

*^1–3^See Dataset S1 for the exact numbers of the genes and more gene sets, which include the gene sets detected in [26] and [28].

*^2^These gene sets were prepared using the data in [29]. In this study, the genes that showed at least 2-fold higher expression with *P-*values <0.05 under the submergence condition than the control condition (non-submergence condition) were defined as up-regulated genes, and the genes that showed at least 2-fold lower expression with *P-*values <0.05 as down-regulated genes.

*^3^This gene set was prepared using the data in [27]. In this study, the genes that showed at least 5-fold higher expression after the 2-hour rehydration treatment preceded by the 2-hour dehydration treatment were defined as up-regulated genes, and the genes that showed at least 2-fold lower expression as down-regulated genes.

*^4^++: *P*>0.99; +: *P*>0.95; **: *P*<0.01; *: *P*<0.05 in cumulative binomial.

### Microarray

A custom microarray which contains approximately 1700 genes of Arabidopsis [30] was provided by Dr. Yoshihiro Narusaka (Research Institute for Biological Sciences, Okayama, Japan). For the microarray analysis, total RNA was extracted from 10-day-old seedlings of wild-type plants and transgenic plants expressing GFP-fused VIP1NLS using RNeasy Plant Mini Kit (Qiagen). cDNA synthesis and hybridization were performed using 3 DNA Array 50 Cy3/Cy5 Kit (Genisphere) following the manufacturer's instructions. Signal detection and normalization (global normalization) were performed at Hokkaido System Science, Japan. Experiments were performed in triplicate and data were averaged. The data are provided as Dataset S3. The genes that showed 2-fold or higher expression levels in the transgenic plants than in the wild type are indicated as up-regulated genes, and the genes that showed 0.5-fold or lower expression levels are indicated as down-regulated genes.

## Results and Discussion

### Functional redundancies among Arabidopsis group I bZIP proteins

**Figure 1:**
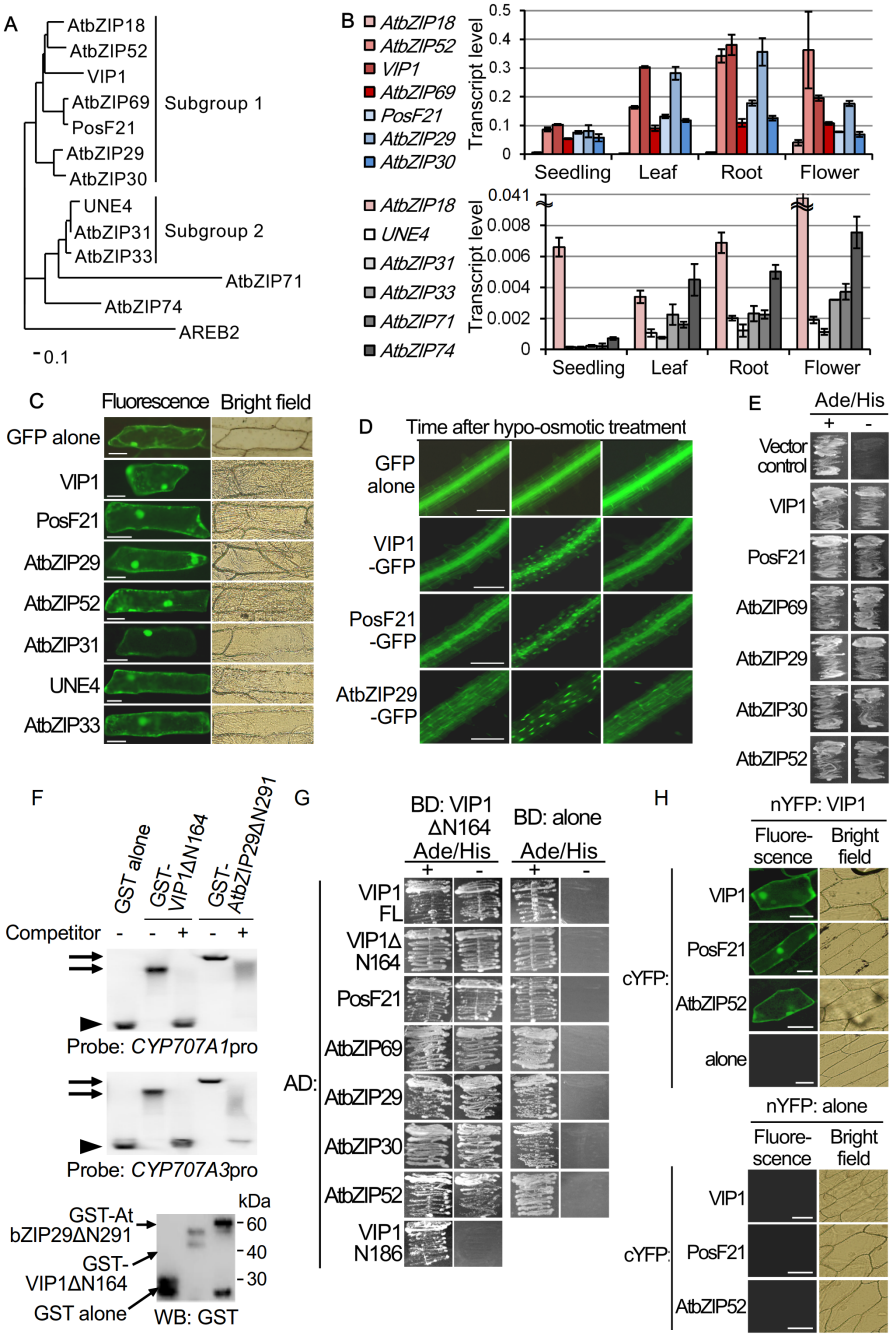
Classification and functional redundancies of Arabidopsis group I bZIP proteins. (A) A rooted phylogenetic tree of the Arabidopsis group I bZIP proteins. The alignment of the amino acid sequences of the group I bZIP proteins (see Fig. S1) was used to generate the distance matrix and the neighbor-joining tree. AREB2 (a group A bZIP protein) was used as an outgroup. (B) Transcript levels of the Arabidopsis group I bZIP protein genes. The absolute amounts of cDNA of the group I bZIP protein genes in the template solution were quantified by qRT-PCR using the standard curve method. The values were normalized by the amount of cDNA of an internal control gene, *UBQ5*, and fold differences from the amount of *UBQ5* cDNA are shown as transcript levels. The expression levels of *AtbZIP18* in the lower panel are the same as those in the upper panel, and shown as control. Data are means of three biological replicates. Error bars indicate SD. (C) Subcellular localization of group I bZIP proteins in onion cells. The indicated proteins were expressed as GFP-fused proteins in onion epidermal cells. For each construct, more than 10 cells were observed, and a representative image is shown. Scale bars  = 100 µm. (D) Subcellular localization of VIP1, PosF21 and AtbZIP29 under a hypo-osmotic condition. GFP-fused VIP1, PosF21 and AtbZIP29 (VIP1-GFP, PosF21-GFP and AtbZIP29-GFP, respectively) were expressed in Arabidopsis. Roots of the transgenic plants were submerged in 20 mM Tris-HCl, pH 6.8 (hypo-osmotic treatment), incubated for 0, 10, 60 minutes, and used for fluorescence microscopy. For each genotype, more than five plants were used for observation, and representative images in the same root are shown. Scale bars  = 100 µm. (E) Transcriptional activation functions of group I bZIP proteins in yeast. The indicated proteins were expressed as GAL4 DNA-binding domain-fused proteins in the yeast reporter strain AH109. Transformed cells were grown on the medium with or without adenine and histidine (Ade/His + or -, respectively). For each construct, three individual colonies were assessed, and a representative result is shown. (F) Interaction between the C-terminal region of AtbZIP29 and *CYP707A1/3* promoters in vitro. The C-terminal regions of VIP1 and AtbZIP29 (the amino acid positions 165–341 for VIP1 and 292–553 for AtbZIP29) were expressed as GST-fused proteins (GST-VIP1Δ164 and GST-AtbZIP29Δ291, respectively) in *E. coli*, purified, and used for the gel shift assay. The presence of GST-VIP1Δ164, GST-AtbZIP29Δ291 and GST alone, which was used as control, in the purified protein solutions was confirmed by Western blotting using an anti-GST antibody (WB: GST, bottom panel). As probes for the gel shift assay, approximately 250-bp DIG-labeled fragments of the *CYP707A1* promoter (*CYP707A1*pro) and the *CYP707A3* promoter (*CYP707A3*pro) were used. As the competitors, the non-DIG-labeled DNA fragments that have the same sequences as the probes were used, and their presence and absence in the reaction mixtures are shown as + and -, respectively (top and middle panels). The experiments were performed three times, and representative results are shown. (G) Interactions between VIP1 and group I bZIP proteins in yeast. The VIP1 variants (VIP1FL: full-length VIP1; VIP1ΔN164: amino acids 165–341 of VIP1; VIP1N186: amino acids 1–186 of VIP1) and the group I bZIP proteins were expressed as GAL4 activation domain-fused proteins (shown as AD:) with GAL4 DNA-binding domain (BD)-fused VIP1ΔN164 (BD: VIP1Δ164) or BD alone (BD: alone) in AH109. VIP1N186 was used as a negative control. Transformed yeast cells were grown on the medium with or without adenine and histidine (Ade/His + or -, respectively). For each combination of the constructs, three individual colonies were assessed, and a representative result is shown. (H) BiFC between VIP1, PosF21 and AtbZIP52 in onion cells. The ORF of *VIP1* was cloned in-frame in front of the coding sequence of the N-terminal region of YFP (nYFP) to express nYFP-fused VIP1 (nYFP: VIP1). The ORFs of *VIP1*, *PosF21*, and *AtbZIP52* were cloned in frame in front of the coding sequence of the C-terminal region of YFP (cYFP) to express the cYFP-fused proteins (shown as cYFP:). These constructs were co-introduced into onion epidermal cells in the indicated combinations. Empty vectors which express nYFP alone and cYFP alone were used for negative controls (shown as nYFP: alone and cYFP: alone, respectively). For each combination, more than 10 cells were observed, and a representative image is shown. Scale bars  = 100 µm.

The locus AT2G13130, which was listed as an Arabidopsis group I bZIP protein gene in an early study [18], was excluded from analyses because it matched a pseudogene in TAIR (The Arabidopsis Information Resource: http://www.arabidopsis.org/). The other 12 members of the Arabidopsis group I protein were further classified into subgroups 1 and 2, and others (AtbZIP71 and AtbZIP74) on the basis of a phylogenetic analysis using their amino acid sequences (Fig. 1A). The absolute transcript levels of the genes encoding these proteins were compared by quantitative RT-PCR (qRT-PCR). In all the tissues studied, the genes encoding the subgroup 1 proteins were more highly expressed than the genes encoding the proteins of the other groups. Among the subgroup 1 protein genes, *VIP1* was the most highly expressed in seedlings, leaves and roots, while *AtbZIP52* was the most highly expressed in flowers (Fig. 1B).

The nuclear-cytoplasmic shuttling of VIP1 is dependent on the nuclear localization signal (NLS) and the nuclear export signal (NES), which are present in the beginning and the end, respectively, of the C-terminal bZIP domain of VIP1 [12], [13], [17]. The NLS, the NES and the bZIP domain are highly conserved among the Arabidopsis group I bZIP proteins (Fig. S1, underlined region). Seven members of the Arabidopsis group I bZIP proteins (VIP1, PosF21, AtbZIP29, AtbZIP52, AtbZIP31, AtbZIP32 and AtbZIP33) were expressed as GFP-fused proteins in onion epidermal cells to examine their subcellular localization. GFP-fused AtbZIP31 showed intense signals in the nucleus, while the other six showed clear signals in the cytosol (Fig. 1C). The number of the amino acids following the putative NES is the smallest in AtbZIP31 among the Arabidopsis group I bZIP proteins (Fig. S1), and this might decrease the activity of the NES of AtbZIP31. Treatment with a hypotonic solution, not with a hypertonic solution, induces transient nuclear accumulation of VIP1 [17]. GFP-fused PosF21 (PosF21-GFP) and GFP-fused AtbZIP29 (AtbZIP29-GFP) were expressed in Arabidopsis (Fig. S2) to examine their subcellular localization under a hypo-osmotic condition. When plants were incubated in a hypotonic solution, the signals of both PosF21-GFP and AtbZIP29-GFP, as well as the signals of GFP-fused VIP1, were first detected mainly in the cytoplasm, then mainly in the nucleus, and then again mainly in the cytoplasm (Fig. 1D), suggesting that the transient nuclear accumulation under the hypotonic condition is somewhat conserved among the group I bZIP proteins. Besides the C-terminal bZIP domain, several conserved sites are present in the N-terminal regions of the group I bZIP proteins (Fig. S1). These conserved sites may be involved in regulating the subcellular localization of the group I bZIP proteins.

The N-terminal region of VIP1 shows a transcriptional activation function in yeast [17]. Transcriptional activation functions of other five members of the Arabidopsis group I bZIP proteins (AtbZIP52, PosF21, AtbZIP69, AtbZIP29, AtbZIP30) were examined by a yeast one-hybrid (Y1H) assay. The bZIP proteins were expressed as GAL4 DNA-binding domain (GAL4BD)-fused proteins in a yeast reporter strain which has a GAL4BD-binding site upstream of the auxotrophic marker genes *ADE2* and *HIS3*. All of the proteins, as well as VIP1, enabled yeast cells to grow on the selection medium lacking adenine and histidine (Fig. 1E), suggesting that these proteins have transcriptional activation functions in yeast.

The bZIP domain is responsible for the DNA-binding ability of bZIP proteins, and the VIP1 C-terminal region alone, which includes the bZIP domain, can bind the *CYP707A1/3* promoters in vitro [17]. In a gel shift assay, the C-terminal region (the amino acid position 292–553) of AtbZIP29 caused a shift in the electrophoretic mobility of the *CYP707A1/3* promoter fragments, and this mobility shift was blocked when an excess amount of competitor DNA was present in the reaction mixture (Fig. 1F), suggesting that AtbZIP29 as well as VIP1 binds the *CYP707A1/3* promoters in vitro. A previous study predicted that plant group I bZIP proteins form homodimers and heterodimers via their bZIP domains [31]. Interactions between VIP1 and other Arabidopsis group I bZIP proteins were examined by a yeast two-hybrid (Y2H) assay and a bimolecular fluorescence complementation (BiFC) assay. In the Y2H assay, six Arabidopsis group I bZIP proteins (VIP1, PosF21, AtbZIP69, AtbZIP29, AtbZIP30 and AtbZIP52) enabled yeast cells to grow on selection medium when co-expressed with the C-terminal region (amino acids 165–341) of VIP1 (VIP1ΔN164), which contains the bZIP domain (Fig. 1G). In the BiFC assay, cYFP (the C-terminal region of yellow fluorescent protein)-fused VIP1, PosF21 and AtbZIP52 all recovered YFP fluorescence when co-expressed with nYFP (the N-terminal region of YFP)-fused VIP1 (Fig. 1H). These results support the above prediction of the dimerization between the bZIP domains of the group I bZIP proteins. bZIP domains are thought to determine the dimerization specificity and the DNA-binding specificity of the bZIP proteins [18], [31]. Together, it is possible that the dimers between VIP1, AtbZIP29 and other group I bZIP proteins have similar DNA-binding specificities, and share target genes.

### Characterization of VIP1-binding sequence as hypo-osmolarity- and/or submergence-responsive element

**Figure 2:**
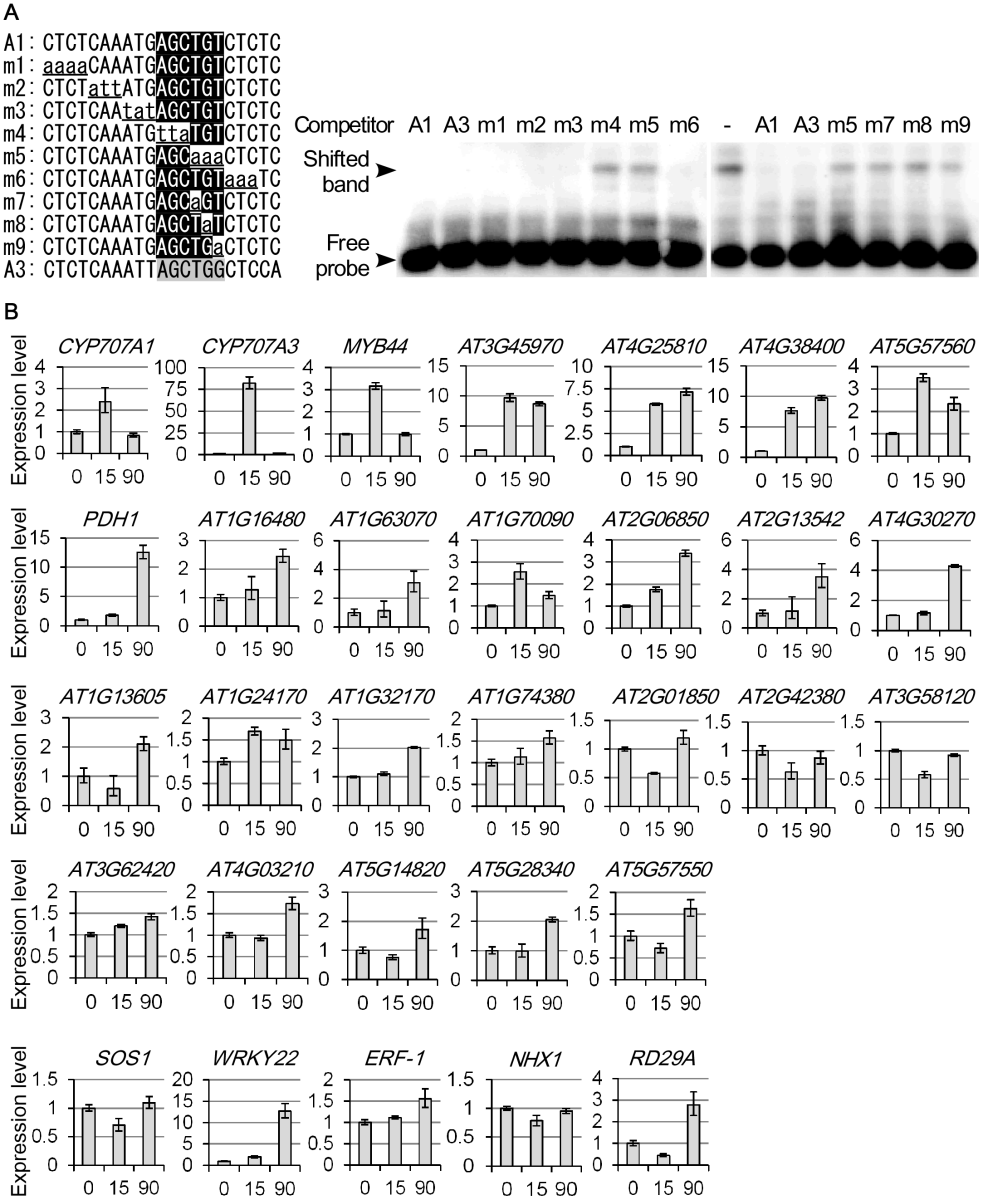
Interaction between VIP1 and AGCTGT/G and its relevance to hypo-osmolarity- and/or submergence-induced gene expression. (A) VIP1 binds AGCTGT/G. The gel shift assay was performed using purified GST-fused VIP1ΔN164 and a DIG-labeled *CYP707A1* promoter fragment as the probe. The 21-bp *CYP707A1/3* promoter fragments (A1 for the *CYP707A1* promoter and A3 for the *CYP707A3* promoter) and point-mutated versions of A1 (m1-9) were used as competitors. In the left panel, sequences mutated from A1 in m1-9 are in lower case and underlined. AGCTGT and AGCTGG in the competitors are indicated by black and gray boxes, respectively. “Competitor -” indicates the absence of the competitors in the reaction mixture. The results of A1 and A3 are shown in both the middle and the right panels for controls. The experiments were performed three times, and a representative result is shown. (B) Quantitative RT-PCR analyses of expression of the genes with promoters containing at least one copy of AGCTGT/G (see Dataset S2 for the exact copy numbers of AGCTGT/G in the promoters of these genes) under a hypo-osmotic condition. Two-week-old wild-type seedlings were submerged in 20 mM Tris-HCl for 0, 15 and 90 minutes, and sampled for RNA extraction and cDNA synthesis. Numbers on x-axes indicate the time points of sampling. Genes up-regulated (>2 fold) either 15 or 90 minutes after the hypo-osmotic treatment are shown in the first two rows. Genes that were not up-regulated by the hypo-osmotic treatment are shown in the third and fourth rows. Genes in the bottom row (*SOS1*, *WRKY22*, *ERF-1*, *NHX1* and *RD29A*) do not have AGCTGT/G in the promoters and are shown as controls. Relative expression levels were calculated by the comparative cycle threshold (C_T_) method using *UBQ5* as an internal control gene. Data are means of three biological replicates. Error bars indicate SD.

In previous studies, VIP1 showed affinity to AGCNGT [15], [32], and a tobacco (*Nicotiana tabacum*) group I bZIP protein, RSG, bound to CAAGCTGG [33], [34]. The *CYP707A1* promoter has AGCTGT at the position −256 to −251 from the start codon, while the *CYP707A3* promoter has a part of the RSG-binding sequence, AGCTGG, at the corresponding position (Fig. S3). By gel shift assays, the VIP1-binding region in the *CYP707A1* promoter was narrowed to approximately 80 bp regions, and these regions contain AGCTGT (Fig. S4). The 21-bp *CYP707A1* promoter fragment containing AGCTGT inhibited the VIP1-dependent band shift when used as competitor in gel shift assays. When AGCTGT was point-mutated, the resultant 21-bp fragment no longer inhibited the VIP1-dependent band shift. The corresponding 21-bp *CYP707A3* promoter fragment and the *CYP707A1* promoter fragment inhibited the VIP1-dependent band shift to the same extent (Fig. 2A). These results suggest that, in agreement with the previous studies, VIP1 has high affinity to AGCTGT or AGCTGG (AGCTGT/G), and that AGCTGT/G is largely responsible for the interactions between VIP1 and the *CYP707A1/3* promoters. RSG, a tomato group I bZIP protein, VSF-1, and rice group I bZIP proteins, Rf2a and Rf2b, can interact with DNA fragments that do not contain AGCTGT/G [33], [35]–[39]. However, those DNA fragments were less effective in inhibiting the VIP1-dependent band shift than either the *CYP707A1/3* promoter fragments or rbe (RSG-binding element), which contains AGCTGG, when used as competitors in gel shift assays (Fig. S5), suggesting that DNA containing AGCTGT/G has higher affinity to VIP1. Even though the *CYP707A1/3* promoter fragments and rbe share AGCTGT/G, the *CYP707A1/3* promoter fragments more effectively inhibited the VIP1-dependent band shift than rbe (Fig. S5, left 4 lanes), suggesting that sequences surrounding AGCTGT/G also affect the interaction between VIP1 and DNA.

Previously microarray analyses were performed to examine rehydration-responsive gene expression [26], [27] and submergence-responsive gene expression [28], [29] in Arabidopsis. In these studies, plants were exposed to hypotonic solutions as either submergence treatment or rehydration treatment. These treatments seemed similar to the treatment performed in our study, thus those microarray data were utilized to evaluate whether AGCTGT/G acts as a hypo-osmolarity-responsive element. Interestingly, when the regions within 500 bp upstream of the start codons were assessed, the proportions of the genes with promoters containing AGCTGT/G (or its complementary sequence C/ACAGCT) were significantly higher in the gene sets which were up-regulated in roots within 0.5–3 hours after the submergence treatment (using a hypotonic solution) and in the gene set which was up-regulated by 2-hour rehydration after 2-hour dehydration than would be expected from the abundance of AGCTGT/G in all the promoters in the whole Arabidopsis genome (Table 1, upper rows). In addition, the proportions of the genes with promoters (500 bp upstream of the start codons) containing AGCTGT/G were significantly lower in the gene sets which were down-regulated 1–12 hours after the root submergence than is expected (Table 1, lower rows). When the promoter regions assessed were extended to 1000 bp upstream of the start codons, the proportions of genes with promoters containing AGCTGT/G were not significantly higher in any gene sets which were up-regulated by the root submergence. However, the proportions of the genes with promoters containing AGCTGT/G were still significantly lower in the gene set which was down-regulated 1–12 hours after the submergence than is expected (Dataset S1). These results raise the possibility that AGCTGT/G within 500 bp upstream of the start codon acts as a cis-element which activates early submergence-responsive genes and that AGCTGT/G within the 1000 bp upstream of the start codon is necessary for maintaining the basal gene expression during the hypo-osmotic condition.

Expression of 26 genes with promoters (500 bp upstream of the start codons) containing at least one copy of AGCTGT/G were examined by qRT-PCR. Fourteen of those genes were more than 2-fold up-regulated either 15 or 90 minutes after the submergence treatment (Fig. 2B). No clear correlation was observed between the numbers of AGCTGT/G in the promoters and the submergence-induced fold changes of the expression of those genes (Dataset S2).

### Generation and characterization of constitutively nuclear-localized variants of VIP1

Because VIP1 variants that have different subcellular localization from the subcellular localization of wild-type VIP1 are thought to be useful to examine the functions of VIP1, several variants of VIP1 were generated and their subcellular localization was examined. First, VIP1 mutants with either Ser → Asp mutation at position 79 (VIP1S79D) or Ser → Ala mutation at position 115 (VIP1S115A) were tested. In previous studies, S79D enhanced the nuclear localization of VIP1 [14], and the mutation corresponding to S115A in tobacco RSG enhanced the nuclear localization of RSG [40], [41]. However, in our experiments, the patterns of the signals of GFP-fused VIP1S79D and GFP-fused VIP1S115A were both similar to the pattern of the signals of GFP-fused wild-type VIP1 (VIP1-GFP) in Arabidopsis treated with a hypotonic solution (Fig. S6), suggesting that neither S79D nor S115A single mutation affects the nuclear-cytoplasmic shuttling of VIP1 under the hypo-osmotic condition. VIP1 has many possible phosphorylation sites according to phosphorylation site-prediction programs (NetPhos: http://www.cbs.dtu.dk/services/NetPhos/, [42]; GPS: http://gps.biocuckoo.org/, [43]), thus multiple phosphorylation sites may be involved in regulating the subcellular localization of VIP1.

**Figure 3:**
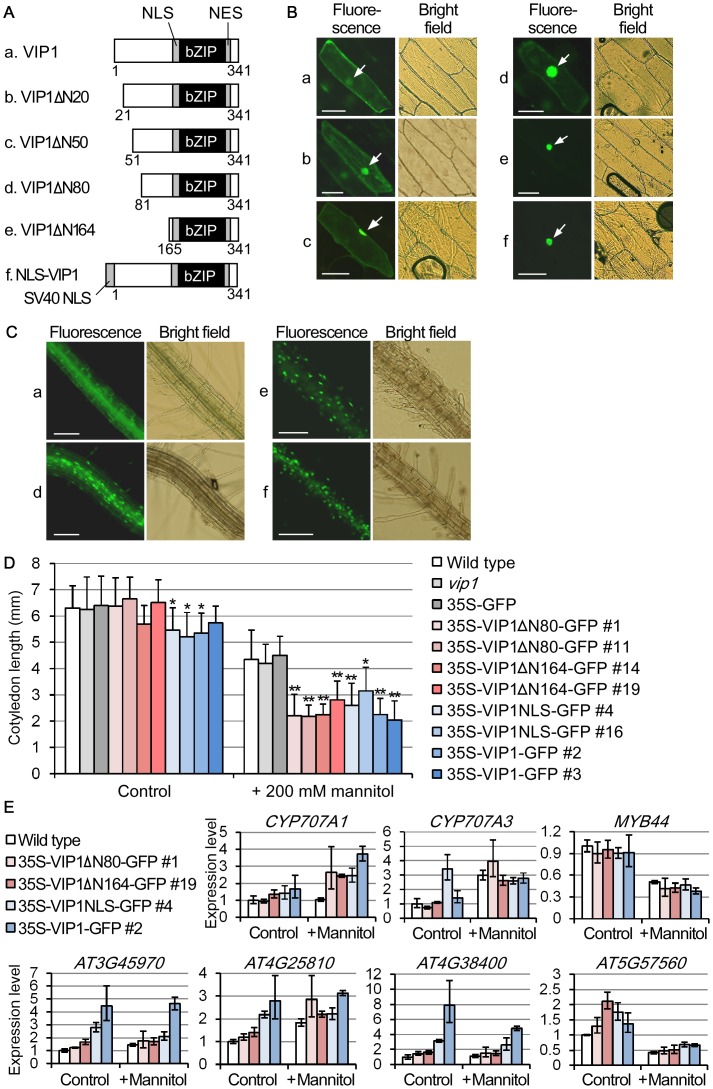
Generation and characterization of constitutively nuclear-localized variants of VIP1. (A) Schematic representations of the VIP1 variants. Numbers indicate amino acid positions of full-length VIP1. Lowercase letters (a–f) correspond to those in the panels B–D. NLS: nuclear localization signal; NES: nuclear export signal. (B) Subcellular localization of the VIP1 variants in onion cells. The indicated forms of VIP1 (a–f) were expressed as GFP-fused proteins in onion epidermal cells. For each construct, more than 10 cells were observed, and a representative image is shown. Scale bars  = 100 µm. (C) Subcellular localization of the VIP1 variants in Arabidopsis. Roots of the transgenic Arabidopsis plants expressing GFP-fused VIP1 variants (forms a, d, e and f) were observed without being treated by a hypotonic solution. For each genotype, more than 10 plants were used for observation, and a representative image is shown. Scale bars  = 100 µm. (D) Expression of the VIP1 variants retards the cotyledon growth under a mannitol-stressed condition. The transgenic plants expressing the GFP-fused VIP1 variants (35S-VIP1ΔN80-GFP, for example) were grown in the presence of 0 (Control) or 200 mM mannitol for 10 days, and their cotyledon lengths were measured. *vip1*, which lacks the 3′ region of the *VIP1* transcript because of a T-DNA insertion in the genomic region of *VIP1* [13], is shown as control. Values are means ± SD. (n = 15–20). *: *P*<0.05; **: *P*<0.01 vs. the wild type in Student's *t*-test. (E) Quantitative RT-PCR analyses of expression of the genes with promoters containing AGCTGT/G in the transgenic plants expressing the VIP1 variants. The transgenic plants were grown in the presence of 0 (Control) or 200 mM mannitol (+ Mannitol) for 18 days, and sampled for RNA extraction and cDNA synthesis. Relative expression levels were calculated by the comparative cycle threshold (C_T_) method using *UBQ5* as an internal control gene. Data are means of three biological replicates. Error bars indicate SD.

Next, N-terminal truncated versions of VIP1 were generated (Fig. 3A). When expressed as GFP-fused proteins in onion epidermal cells, the N-terminal 20 amino acid-truncated version of VIP1 (VIP1ΔN20, form b) showed clear cytoplasmic signals similar to the signals of VIP1-GFP (Fig. 3B, panels a and b), but the 50 amino acid- and 80 amino acid-truncated versions (VIP1ΔN50 and VIP1ΔN80, forms c and d, respectively) showed weaker cytoplasmic signals and intense nuclear signals (Fig. 3B, panels c and d). The signals for the GFP-fused 164 amino acid-truncated version (VIP1ΔN164, form e) were almost limited to the nucleus (Fig. 3B, panel e). These results suggest that the N-terminal truncations enhance the nuclear localization of VIP1.

SV40 (Simian virus 40) NLS functions as an active NLS in plants [44]. The signals of a VIP1 variant which has SV40 NLS, which functions in the N-terminus (VIP1NLS, Fig. 3A, form f) were detected only in the nucleus in onion cells when it was expressed as GFP-fused protein (Fig. 3B, panel f), suggesting that the N-terminal fusion of a functional NLS can convert VIP1 into a constitutively nuclear-localized protein.

In a Y1H assay, VIPΔN80 enabled yeast cells to grow on the selection medium when expressed as a GAL4BD-fused protein, while VIP1ΔN164 did not (Fig. S7), raising the possibility that these VIP1 variants have different effects on the expression of VIP1 target genes. VIP1ΔN80, VIP1ΔN164 and VIP1NLS were expressed as GFP-fused proteins (VIP1ΔN80-GFP, VIP1ΔN164-GFP and VIP1NLS-GFP, respectively) in Arabidopsis (Fig. S8) to further examine the physiological roles of VIP1 and group I bZIP proteins. In agreement with the results of the transient expression experiments using onion cells (Fig. 3B), in Arabidopsis, VIP1ΔN80-GFP, VIP1ΔN164-GFP and VIP1NLS-GFP all showed intense nuclear signals even when cells were not treated with a hypotonic solution (Fig. 3C). In a previous study, overexpression of VIP1-GFP did not affect the survival rate of plants that were dehydrated and then rehydrated, but it retarded plant growth in the presence of high concentrations of mannitol. In the same study, VIP1-GFP was localized mainly in the cytoplasm under the mannitol-stressed conditions [17]. In the presence of 200 mM mannitol, cotyledon growth was more severely inhibited in transgenic plants expressing VIP1-GFP, VIP1ΔN80-GFP, VIP1ΔN164-GFP and VIP1NLS-GFP than in the wild-type plants (WT) and the other control plants (Fig. 3D). The amount of VIP1 in the nucleus may be higher in VIP1-GFP-overexpressing plants than in WT, and this may be sufficient to cause the phenotype similar to the phenotypes caused by the expression of the constitutively nuclear-localized variants of VIP1. Alternatively, the expression of VIP1 itself rather than its nuclear localization might be responsible for causing the growth-retardation phenotype under the mannitol-stressed condition. Because VIP1ΔN80 and VIP1ΔN164, which showed different transcriptional activation functions in yeast (Fig. S8), caused the similar phenotype in the mannitol-stressed plants, the putative transcriptional activation domain of VIP1 may not be involved in regulating the mannitol responses. *CYP707A1/3*, which play roles in ABA catabolism [3]–[5], are two putative target genes of VIP1 [17], but no significant difference was observed between WT and the transgenic lines in ABA-induced inhibition of seed germination (Fig. S9).

In qRT-PCR, four out of the seven submergence-induced genes with promoters containing AGCTGT/G were more highly expressed in either the presence of 200 mM mannitol or its absence in the transgenic plants overexpressing VIP1-GFP and VIP1NLS-GFP than in WT. However, the expression levels of these genes were just slightly higher (less than 2 fold) in the transgenic plants overexpressing VIP1ΔN80-GFP, VIP1ΔN164-GFP than in WT (Fig. 3E, *CYP707A1*, *AT3G45970*, *AT4G25810* and *AT4G38400*). In addition, a larger-scale gene expression analysis using a custom microarray containing approximately 1700 Arabidopsis genes [30] detected only 10 genes down-regulated (more than 2-fold) in the VIP1NLS-GFP-expressing plants, and one gene up-regulated. No clear correlation was observed between these genes and either AGCTGT/G in the promoters or the submergence-responsive genes in the previously published microarray data (Dataset S3), suggesting that complicated mechanisms still underlie in regulating the hypo-osmolarity and/or submergence-responsive gene expression. VIP1 and other group I bZIP proteins can bind not only AGCTGT/G but also other DNA sequences (Fig. S5), and these non-AGCTGT/G sequences might also be involved in the hypo-osmolarity and/or submergence-responsive gene expression. It may also be possible that constitutively localizing VIP1 to the nucleus is not sufficient to fully regulate the expression of VIP1 target genes. For example, an Arabidopsis group A bZIP protein, AREB1, is constitutively nuclear-localized, but requires phosphorylation to fully activate its target genes [45]. In addition, Arabidopsis group C bZIP proteins, AtbZIP10 and AtbZIP25, form heterodimers with a group S bZIP protein, AtbZIP53, and the heterodimers interact with a transcriptional coactivator, ABI3, to enhance the target gene activation [46]. As in these examples, even if localized in the nucleus, VIP1 may require post-translational modifications and/or protein-protein interactions to fully control the expression of its target genes.

In conclusion, our current data support the idea that VIP1 and the other group I bZIP proteins, especially those having higher expression levels, are similar in the subcellular localization, the transcriptional activation function and the DNA-binding specificity, and that the VIP1-binding sequence AGCTGT/G has a role in regulating the submergence-responsive gene expression. Further studies are required to elucidate the physiological roles as well as the above-described cytological and biochemical functions of the group I bZIP proteins.

## Supporting Information

Figure S1 **Alignment of the amino acid sequences of Arabidopsis group I bZIP proteins.**
(PDF)

Figure S2 **An RT-PCR analysis of transcripts of** ***GFP*** **-fused** ***PosF21*** **and** ***GFP*** **-fused** ***AtbZIP29*** **in Arabidopsis.**
(PDF)

Figure S3 **Alignment of the sequences of the** ***CYP707A1/3*** **promoters.**
(PDF)

Figure S4 **Gel shift assays for narrowing the VIP1-binding region in the** ***CYP707A1*** **promoter.**
(PDF)

Figure S5 **The** ***CYP707A1/3*** **promoter fragments most effectively inhibit the VIP1-dependent band shift.**
(PDF)

Figure S6 **Subcellular localization of VIP1 variants that have point mutations in the putative phosphorylation sites.**
(PDF)

Figure S7 **Transcriptional activation function of the truncated versions of VIP1 in yeast.**
(PDF)

Figure S8 **An RT-PCR analysis of expression of transcripts for GFP-fused VIP1 variants in Arabidopsis.**
(PDF)

Figure S9 **Expression of VIP1 variants do not affect ABA-induced inhibition of seed germination.**
(PDF)

Table S1 **cDNA clones obtained from RIKEN.**
(PDF)

Table S2 **Primers used for qRT-PCR analyses of expression of Arabidopsis group I bZIP protein genes.**
(PDF)

Table S3 **Primers used for semi-quantitative RT-PCR analyses of transgene expression.**
(PDF)

Table S4 **Primer pairs used to generate constructs for Y1H and Y2H assays.**
(PDF)

Table S5 **Primer pairs used to generate constructs for expression of nYFP-, cYFP- and GFP-fused proteins.**
(PDF)

Table S6 **Primers used to generate the point-mutated versions and the NLS-attached version of VIP1.**
(PDF)

Dataset S1 **The numbers and the proportions of the genes with promoters containing AGCTGT/G in the submergence-responsive genes.**
(XLS)

Dataset S2 **Complete list of the genes with promoters containing AGCTGT/G within 500 bp upstream of the start codons and summary of RT-PCR analyses using some of them.**
(XLS)

Dataset S3 **Data and summary of the microarray analysis.** distributions using 0.185 and 0.033 (values in the first row) as the probabilities.
(XLS)
