# Supplementary material for: Analysis of Functions of VIP1 and Its Close Homologs in Osmosensory Responses of Arabidopsis thaliana
Source: PLoS One. 2014 Aug 5;9(8):e103930. doi: 10.1371/journal.pone.0103930 (PMC4122391; doi:10.1371/journal.pone.0103930)
Supplement: Figure S1 — Alignment of the amino acid sequences of Arabidopsis group I bZIP proteins. (PDF) [file pone.0103930.s001.pdf]

Supporting figures and tables

```
bZIP18   1   ------------------------------------------------------------------------------ 1
bZIP52   1   ------------------------------------------------------------------------------ 1
VIP1     1   ------------------------------------------------------------------------------ 1
bZIP69   1   ------------------------------------------------------------------------------ 1
PosF21   1   ------------------------------------------------------------------------------ 1
bZIP29   1   MG-DTEKCNSDMIQRLHSSFGTTSSSIPKNPISQLDLNPNFIRSSAPQFSKPFSDSGKRIGVPPSHPNLIPPTSPFSQIP 79
bZIP30   1   MGGGGDTTDTNMMQRVNSSSGTSSSSIPKH---NLHLNPALIR-SHHHFRHPFT------GAPPPP---IPPISPYSQIP 67
UNE4     1   ------------------------------------------------------------------------------ 1
bZIP31   1   ------------------------------------------------------------------------------ 1
bZIP33   1   ------------------------------------------------------------------------------ 1
bZIP74   1   -----MNGRGNMTQYQQNPFSTDDGGQSTGVSLSSRTSLSPPLIRYPAGSPDFSPGPRCTTQPSPTFSDFTQASPSLTSF 75
bZIP71   1   ------------------------------------------------------------------------------ 1

bZIP18   1   ---------------MEDPSNPQPNQSNLSQCPP---------------------------------------LATAPT 25
bZIP52   1   ---------------MEKSDPPPVPKPGATIIPS---------------------------------------SDPIPN 25
VIP1     1   --------------MEGGGRGPNQTILSEIEHMP--------------------------------------------- 20
bZIP69   1   ---------------MDKEKSPAPPPSGGLPPPS--GRYSAFSPNGSSFAMKAESSFPPLTPSGSNSSDANRFSHDISRM 63
PosF21   1   ---------------MDKEKSPAPP-CGGLPPPSPSGRCSAFSEAG---------------PIG-HGSDANRMSHDISRM 48
bZIP29   80  TTRQPGSHNFNPGGANHSRSMSQPNSFFSFDSLPPLSPSPFRDHDVSMEDRDSGVFNSNHSLPPSPFTRCNSTSSSSLRV 159
bZIP30   68  ATLQP----------RHSRSMSQPSSFFSFDSLPPLNPS-APSVSVSVEEKTGAGFSP--SLPPSPFTMCHSSSSRNAGD 134
UNE4     1   --------------MNGSDN-TTPSRPWSITQPS----------------------------------------LAFSS 24
bZIP31   1   --------------MNGSDN-STPSRPRSITQPS----------------------------------------LAFSS 24
bZIP33   1   --------------MNGSDNITTPSGSRSMTQPS----------------------------------------LASSS 25
bZIP74   76  NNPASFTPSFSFSNIHQMIPTPSSSHNSKASVSSASSSSFYFPQTSPSSCSTPSSFSPDSFSHSNTGPWSIPQPSPVFSS 155
bZIP71   1   ----------------MTISYPAKEFLQTIFKTPYLKN-----------------------------------LYLSPTMSI 31

bZIP18   26  PAPVRG-PYHRRAHS-------------------EVQFRLPEDLDLS----------EPFGGFDELG------SEDDLFC 69
bZIP52   26  ADPIPSSSFHRRSRSDD-----------------MSMFMFMDPLSSA----------APPS-SDDLP-----SDDDLFS 71
VIP1     21  EAPRQRISHHRRARSETFFSGESID-----------DLLLFDPSDIDFSSLDFLNAPPPPQQSQQQPQASPMSVDSEETS 89
bZIP69   64  PDNPPKNLGHRRAHS--------------------EILTLPDDLSFDSDLGVVG-AADGPSFSDD--------TDEDLLY 114
PosF21   49  LDNPPKKIGHRRAHS--------------------EILTLPDDLSFDSDLGVVGNAADGASFSDE--------TEEDLLS 100
bZIP29   160 GESLPPRKSHRRSNSDIPSGFNSMP-----LIPPRPLERSFSGGECADWSKSNPFVKKESSCEREGVGER--EAMDDLFS 232
bZIP30   135 GENLPPRKSHRRSNSDVTFGFSSMMSQNQKSPPLSSLERSISGEDTSDWSNLVKKEPREGFYKGRKPEVE--AAMDDVFT 212
UNE4     25  LPPLSPSPSSSRRNS-------------------LPLMN--PSTSME----------SRDSSMRFK---------KNSLLP 65
bZIP31   25  LPPLSPSPSSSRRNS-------------------IPLMN--PSASVE----------SRDSSIRVK---------KKSLLP 65
bZIP33   26  LPPLSPSSSTFRRNS-------------------LPSMSFSPSAPVE----------SRDSSIKVN---------KNPSLP 68
bZIP74   156 IAPASSALSSFGPDSFSHSNTGTWSIP-----QPSPVFSSIAPASSAPPLFGRDSFPRSNRGKGSLIHRS---PVLSVLP 227
bZIP71   32  YDTLIGSFATTLKRYLLKS----------------DVAVMYTSPSTYVE----------NEDSSFRFN--------QNCSPMP 80

bZIP18   70  SYMDIE----------------------------KLGSGSGSASDSAGPSAPRSDNP---------FSAENGGAEAGNSR 112
bZIP52   72  SFIDVD----------------------------SLTSNPNPFQNPS-----LSSNS---------VSGAANPPPPPSSR 109
VIP1     90  SNGVVP----------------------------PNSLPPKPEARFGRHVRSFSVDSDFFDDLGVTEEKFIATSSGEKKK 141
bZIP69   115 MYLDME----------------------------KFNSSATSTSQMGEPSEPTWRNE------LASTSNLQSTPGSSSER 160
PosF21   101 MYLDMD----------------------------KFNSSATSSAQVGEPSGTAWKNETMMQTGTGSTSNPQNTVNSLGER 152
bZIP29   233 AYMNLENIDVLNSSEADDSKNGNENRDDMES-SRASGTKTNGSDTEGESSSVNESANNNMNSSGEKRESVKRRAAGGDIA 311
bZIP30   213 AYMNLDNIDVLNSFGGEDGKNGNENVEEMES-SRGSGTKKTNGGSSSDSE-GDSSASGNVKVALSSSSSGVKRRAGGDIA 290
UNE4     66  PLGVKK--------------------------IAPKD-IGPLKRHYRSVSMDSCLSDLLKLTPSPGNTPSSRLVDGDQNA 118
bZIP31   66  PLCVKR--------------------------RAPKDDIEPFKRHYRSLSVDSCLSDLLKLPPSPNNVSSSRSVDGEQNA 119
bZIP33   69  PL--------------------------------------------------------------------VDGEEDA 77
bZIP74   228 PAPVYSS--------------------------NPMVRSSPPGHPSPSAHLEEMSNRPPLHPQPRVPVTRSNSAKVSGSR 281
bZIP71   81  PWVLG------------------------------TKSKTIINTLPPKKSHKRTNTDIIGIHSMTSQNSSGSSYFQLLD 129

bZIP18   113 P--RHRHSLSVDGS-------------------------------------------STLES---IEAKKAMAPDKIAEL 144
bZIP52   110 P--RHRHSNSVDAG-------------------------------------------CAMYAGDIMDAKKAMPPEKLSEL 144
VIP1     142 GNHHHSRSNSMDGEMSSAS-------------------------------FNIESIIASVSGKDSGKKNMGMGGDRIAEL 190
bZIP69   161 PRIRHQHSQSMDGSTTIKP----------------------------------EMLMSGNEDVSGVDSKKAISAAKLSEL 206
PosF21   153 PRIRHQHSQSMDGSMNIN-----------------------------------EMLMSGNEDDSAIDAKKSMSATKIAEL 197
bZIP29   312 PTTRHYRSVSVDSCFMEKLSFGDES-LKPPPSPGSMSRKVSPTNSVDGNSGAAFSIEFNNGEFTAAEMKKIMANDKIAEM 390
bZIP30   291 PTGRHYRSVSMDSCFMGKLNFGDESSLKLPPSS---SAKVSPTNSGEGNS-SAYSVEFGNSEFTAAEMKKIAADEKIAEI 366
UNE4     119 SRLEFDANDYTDDELN---------------------------------------------------KIAKSNKLKEV 145
bZIP31   120 SELEFDTSVYTDDELN---------------------------------------------------KIAKSTKLKEV 146
bZIP33   78  SWLEFGSSDYTDDELN----------------------------------------KIAKSTKLQDIAKSTKLQEI 113
bZIP74   282 PRKYHKRTNSELSSMLVGDSSRGEEGGFGKLIYN------EEAMKEFCSEYMIMPNQSAVNNSDQNRNADVLMITNTDSG 355
bZIP71   130 LLADLGELIYDDEAMKG---------------------------------FYYEFMNLDNKNAFNNDENLNNLMITFT 174
```

```
bZIP18   145 WVVDPKRAKR------IIANRQSAARSKERKAR---YILELERKVQTLQTEATTLSAQLSLFQRDTTGLSSENTELKLRL 215
bZIP52   145 WNIDPKRAKR------IIANRQSAARSKERKAR---YIQELERKVQSLQTEATTLSAQLTLYQRDTNGLANENTELKLRL 215
VIP1     191 ALLDPKRAKR------IIANRQSAARSKERKIR---YTGELERKVQTLQNEATTLSAQVTMLQRGTSELNTENKHLKMRL 261
bZIP69   207 ALIDPKRAKR------IWANRQSAARSKERKMR---YIAELERKVQTLQTEATSLSAQLTLLQRDTNGLGVENNELKLRV 277
PosF21   198 ALIDPKRAKR------IWANRQSAARSKERKTR---YIFELERKVQTLQTEATTLSAQLTLLQRDTNGLTVENNELKLRL 268
bZIP29   391 AMSDPKRVKRNDPLFRIIANRQSAARSKERKMR---YIVELEHKVQTLQTEATTLSAQLTLLQRDMMGLTNQNNELKFRL 467
bZIP30   367 VMADPKRVKR------IIANRVSAARSKERKTR---YMAELEHKVQTLQTEATTLSAQLTHLQRDSMGLTNQNSELKFRL 437
UNE4     146 AL-DPKEVRR------ILKNRESAAHLKQKKLQ---YMINLEHRINFVENENASIFEKIKLLENDKTMMMNEKKEIMIRI 215
bZIP31   147 AS-DPKEVRR------ILKNQESAARSKQKKLQ---YMINLELKINFLENKNASIFEKIKLLENDKTMRMNEKKEIMIRI 216
bZIP33   114 VS-DPKKVRR------ILKNREIAASSKQRKLK---YMIDLEHRIKFLENKNALIFEKIKLLEKDKTILMNEKKEITIQI 183
bZIP74   356 GANDAKKYKR------MLANRASAARSKENREK---KIRDMELRVETLENTQASLFGTMTLLEKENIVMMNENKLAKIRL 426
bZIP71   175 NLGGASNANESG---LKIGATTSGVKTKVDEDI---EPLCRHYKCATMDSSFIHWITHWNLLDQVFSSNFVGEGEYYTDP 248

bZIP18   216 QVMEQQAKLRD------ALNEQLKKEVERLKFATGEVS-PADAYNLGMAHMQYQQQPQQSFFQHHHQQQTDAQNLQQMTH 288
bZIP52   216 QAMEQQAQLRN------ALNEALRKEVERMKMETGEISGNSDSFDMGMQQIQYSSSTFMAIPPYHGSMN---LHDMQMHS 286
VIP1     262 QALEQQAELRD------ALNEALRDELNRLKVVAGEIPQGNGNSYNRAQFSSQQSAMNQFGNKTNQQMSTNGQPSLPSYM 335
bZIP69   278 QTMEQQVHLQD------ALNDALKEEVQHLKVLTGQGPSNGTSMNYGS-----FGSNQQ-FYPNNQSMHTILAAQQLQQL 345
PosF21   269 QTMEQQVHLQD------ELNEALKEEIQHLKVLTGQVAP--SALNYGS-----FGSNQQQFYSNNQSMQTILAAKQFQQL 335
bZIP29   468 QAMEQQARLRD------ALNEALNGEVQRLKLAIGESSQNESERSKMQS---LNAEMFQQLNISQLRQQPQQMQQQSHQQ 538
bZIP30   438 QAMEQQAQLRDGMHIIKTLSEKLNEEVQRLKLVIGEPNRRQSGSSSSESKMSLNPEMFQQLSISQLQH--QQMQHS---- 511
UNE4     216 ESMEIQAQLRD------VLTEHLHGESERLKAALISNENGNGKVQKLRMATCEVLQNRHEFDKSNMEVMDSNMINWSQPN 289
bZIP31   217 ESLEQHAELRD------ALTEHLHVEIERLKAVLISNEKGNEKSPKSR--------------------DDNM------- 262
bZIP33   184 ESLEQQAQLRD------ALTEKLHVEIERLKVITISNEKGSVELQRLKMETCEVLQYRREFDRSNMQGMDPNMFTWSQPN 257
bZIP74   427 QLLEQQAPLLT------ALTKQLDELRRLEKEANERGSVDYSQLLKQLKQSEELLIAEINRFKVATGQGMKNPNQFEGSTM 500
bZIP71   249 NEVKRILENRD--------LDTRSKQGKSLYIVDLEGSVRIHVMVNTSLYGMIVLLEVRHITLAKKKNDVDDEREQENED 320

bZIP18   289 QFHLFQPNNNQNQSSRTNPPTAHQLMHHATSNAPAQSHSYSEAMHEDHLGRLQGLDISSCGR--GSNFGRSDTVSESSST 366
bZIP52   287 SFNPMEMSNSQ---------------------------SVSDFLQN---GRMQGLEISSNSSSLVKSEGPSLSASESSSA 336
VIP1     336 DFTKRG-------------------------------------------------------------------------- 341
bZIP69   346 QIQSQKQQQQQQQHQQ---QQQQQ-----QQQFHFQQQQLYQLQQQQ---RLQQQEQQ-SGASELRRPMPSPGQKESVTS 413
PosF21   336 QIHSQKQQQQQQQQQQQHQQQQQQ-----QQQYQFQQQQMQQLMQQ----RLQQQEQQ-NGV----RLKPSQAQKEN--- 398
bZIP29   539 NHQNGTMATKSESNE----------------------------------------------------------------- 553
bZIP30   511 -NQCSTMKAKHTSND----------------------------------------------------------------- 525
UNE4     290 PGFNG--------------------------------------------------------------------------- 294
bZIP31   262 -------------------------------------------------------------------------------- 262
bZIP33   258 LGFYGQI------------------------------------------------------------------------- 264
bZIP74   501 HQSDQNVFQPQLNTYEFNQHQQLDP------NIFKEQCNVNEFNHEQPNHEFYGHN------------------------ 550
bZIP71   321 SS------------------------------------------------------------------------------ 322

bZIP18   367 M--------- 367
bZIP52   337 Y--------- 337
VIP1     341 ---------- 341
bZIP69   414 PDRETPLTKD 423
PosF21   398 ---------- 398
bZIP29   553 ---------- 553
bZIP30   525 ---------- 525
UNE4     294 ---------- 294
bZIP31   262 ---------- 262
bZIP33   264 ---------- 264
bZIP74   550 ---------- 550
bZIP71   322 ---------- 322
```

Figure S1. Alignment of the amino acid sequences of Arabidopsis group I bZIP proteins. The amino acid sequences were aligned using Clustal W [47]. Identical amino acid residues conserved in more than four sequences are boxed in dark gray. Similar amino acid residues conserved in more than four sequences are boxed in light gray. The region corresponding to the bZIP domain of VIP1 is shown by the solid underline. The regions corresponding to the NLS and the NES of VIP1 are shown by the dashed underline and the dotted underline, respectively. The bZIP domain, the NLS and the NES of VIP1 were predicted using ELM (Eukaryotic Linear Motif resource: http://elm.eu.org/, [48]). The serine residues at positions 79 and 115 in VIP1 are indicated as *.

Supporting references

47. Thompson JD, Higgins DG, Gibson TJ (1994) CLUSTAL W: improving the sensitivity of progressive multiple sequence alignment through sequence weighting, position-specific gap penalties and weight matrix choice. Nucleic Acids Res 22: 4673-4680.

48. Dinkel H, Michael S, Weatheritt RJ, Davey NE, Van Roey K, et al. (2012) ELM--the database of eukaryotic linear motifs. Nucleic Acids Res 40: D242-251.
