# Supplementary material for: Analysis of Functions of VIP1 and Its Close Homologs in Osmosensory Responses of Arabidopsis thaliana
Source: PLoS One. 2014 Aug 5;9(8):e103930. doi: 10.1371/journal.pone.0103930 (PMC4122391; doi:10.1371/journal.pone.0103930)
Supplement: Figure S2 — An RT-PCR analysis of transcripts of GFP -fused PosF21 and GFP -fused AtbZIP29 in Arabidopsis. (PDF) [file pone.0103930.s002.pdf]

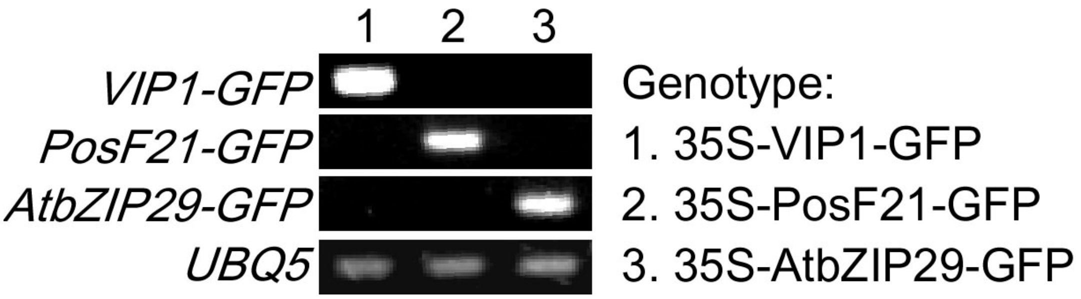

Figure S2. An RT-PCR analysis of transcripts of *GFP*-fused *PosF21* and *GFP*-fused *AtbZIP29* in Arabidopsis. Transgenic plants expressing GFP-fused VIP1, GFP-fused PosF21 or GFP-fused AtbZIP29 (35S-VIP1-GFP, 35S-PosF21-GFP or 35S-AtbZIP29-GFP, respectively) were grown for 10 days, and sampled for RNA extraction and cDNA synthesis. The expression of *UBQ5* is shown as control. The lane numbers correspond to the genotype numbers. Experiments were performed three times and a representative result is shown.
