# Supplementary material for: Analysis of Functions of VIP1 and Its Close Homologs in Osmosensory Responses of Arabidopsis thaliana
Source: PLoS One. 2014 Aug 5;9(8):e103930. doi: 10.1371/journal.pone.0103930 (PMC4122391; doi:10.1371/journal.pone.0103930)
Supplement: Figure S3 — Alignment of the sequences of the CYP707A1/3 promoters. (PDF) [file pone.0103930.s003.pdf]

```
TTTGGCAACA-----GAAC--GAATTA-----TTAACCAAATTAATCCAT--TTGAATGGCCCTCCTCTCTCT--CT
TTTGTCTATACTTAAGGGCCAAAATTGCCCCTTTCCTTGACTTAATCCATAATTAATTAAAGAGGCATAATTTAATT

GTCTTTAATTAGTTGGTTTGGTCAAAATCTCACACGCTC--------TTTATGCATGCTCTTTGACACGTGCTATCT
AACGGTGGCTAAATT-TTTGGTCAAAATCTCACACGCTCCTTTTTTTTTTTTGCATGCTCTTGGACACGTGCTCTCG

TCG-TCTCTCTCAAATGAGCTGTCTCTCTAATAAGAGTTTCTTCTGTATAGGAAAGGAA---AGGTCTCTCTCTCTC
TCACTCTCTCTCAAATTAGCTGGCTCC--AAAAAAGGTTCCTTTTAGGAAACTCCCTTCTCCAATTTGTTTCCTCAT

TATTTACTTATGAGAATAGCCTATAAAACCCCTTCACATCTCCCACTTG-TTTTGCATCACAAACAT-----TTCTT
CCTTCTCTCTCCTCCACTGCCTATAAAACCAATTCATCTCTCCCACTTGATTTTTCATCTCCAACACAAAAACCAAT

CCATTACATTAAAACTCCAAAAAATTCATTTTTGTTTTCTTTTAGAGTTCACAAGTTCTTCGTTGTTCAGCTACTCC
CCATTAGAGAGAGAACTCACAAAACATACTTCGAATTCCCATT---GTTTAAAAGACGAAGATA-------------

CACTGTCATAACACGAAGTGGGTTTTTTTCTGATCAAAGAACAAAAACAAAA
----------------------------------------------------
```

Figure S3. Alignment of the sequences of the *CYP707A1/3* promoters. The sequences of the *CYP707A1* promoter and the *CYP707A3* promoter correspond to the upper row and lower row, respectively. Approximately 350-bp sequences upstream of the start codon were aligned. Gray boxes indicate DNA residues shared between the promoters. AGCTGT and AGCTGG are highlighted by rectangles. Black boxes indicate putative transcriptional initiation positions. The sequence of the probes for the gel shift assays in Fig. 1F, Fig. 2A and Fig. S5 is underlined.
