# Supplementary material for: Analysis of Functions of VIP1 and Its Close Homologs in Osmosensory Responses of Arabidopsis thaliana
Source: PLoS One. 2014 Aug 5;9(8):e103930. doi: 10.1371/journal.pone.0103930 (PMC4122391; doi:10.1371/journal.pone.0103930)
Supplement: Figure S4 — Gel shift assays for narrowing the VIP1-binding region in the CYP707A1 promoter. (PDF) [file pone.0103930.s004.pdf]

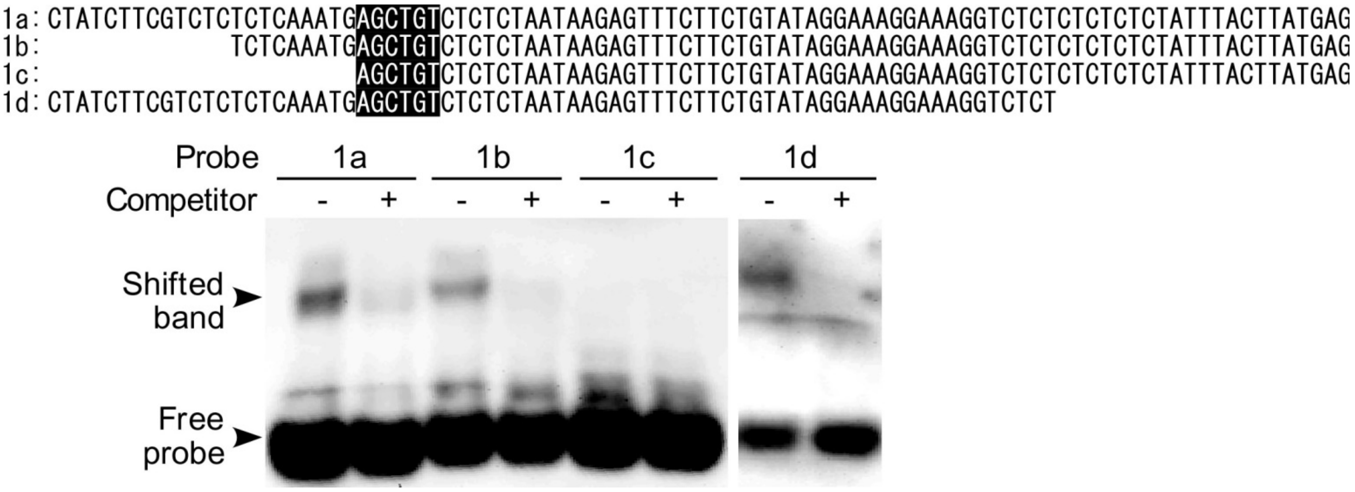

Figure S4. Gel shift assays for narrowing the VIP1-binding region in the *CYP707A1* promoter. Partial fragments of the *CYP707A1* promoter (1a-d) were labeled by DIG and used as probes. Non-labeled fragments of the same regions were used as competitors. The presence and absence of the competitors in reaction solutions are shown as + and -, respectively. Experiments were performed three times, and representative results are shown.
