# Supplementary material for: Analysis of Functions of VIP1 and Its Close Homologs in Osmosensory Responses of Arabidopsis thaliana
Source: PLoS One. 2014 Aug 5;9(8):e103930. doi: 10.1371/journal.pone.0103930 (PMC4122391; doi:10.1371/journal.pone.0103930)
Supplement: Figure S5 — The CYP707A1/3 promoter fragments most effectively inhibit the VIP1-dependent band shift. (PDF) [file pone.0103930.s005.pdf]

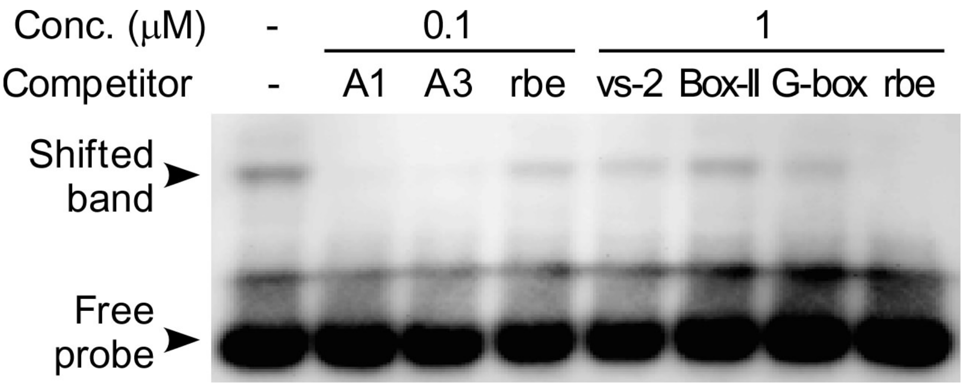

Figure S5. ***CYP707A1/3*** **promoter fragments most effectively inhibit the VIP1-dependent band shift.** The gel shift assay was performed using a *CYP707A1* promoter fragment as the probe. Seven different oligonucleotides that are known to interact with plant group I bZIP proteins (*CYP707A1/3* promoter fragments (designated as A1 and A3) for Arabidopsis VIP1; rbe for tobacco RSG; vs-2 for tomato VSF-1; Box-II for rice Rf2a and Rf2b; G-box for RSG) were used as competitors. The concentrations of the competitors in reaction solutions (either 0.1 or 1 μM) are indicated. Experiments were performed three times and a representative result is shown. The oligonucleotide sequences used are: A1: 5'-CTCTCAAATGAGCTGTCTCTC-3'; A3: 5'-CTCTCAAATTAGCTGGCTCCA-3'; rbe: 5'-CCCCAAAGTCCAGCTTGAAAT-3'; vs-2: 5'-CATGCTCCGTTGGATGT-3'; Box-II: 5'-CCAGTGTGGCGCTGG-3'; G-box: 5'-TCAGACACGTGGCATG-3' (only forward sequences are shown for each).
