# Supplementary material for: Analysis of Functions of VIP1 and Its Close Homologs in Osmosensory Responses of Arabidopsis thaliana
Source: PLoS One. 2014 Aug 5;9(8):e103930. doi: 10.1371/journal.pone.0103930 (PMC4122391; doi:10.1371/journal.pone.0103930)
Supplement: Figure S6 — Subcellular localization of VIP1 variants that have point mutations in the putative phosphorylation sites. (PDF) [file pone.0103930.s006.pdf]

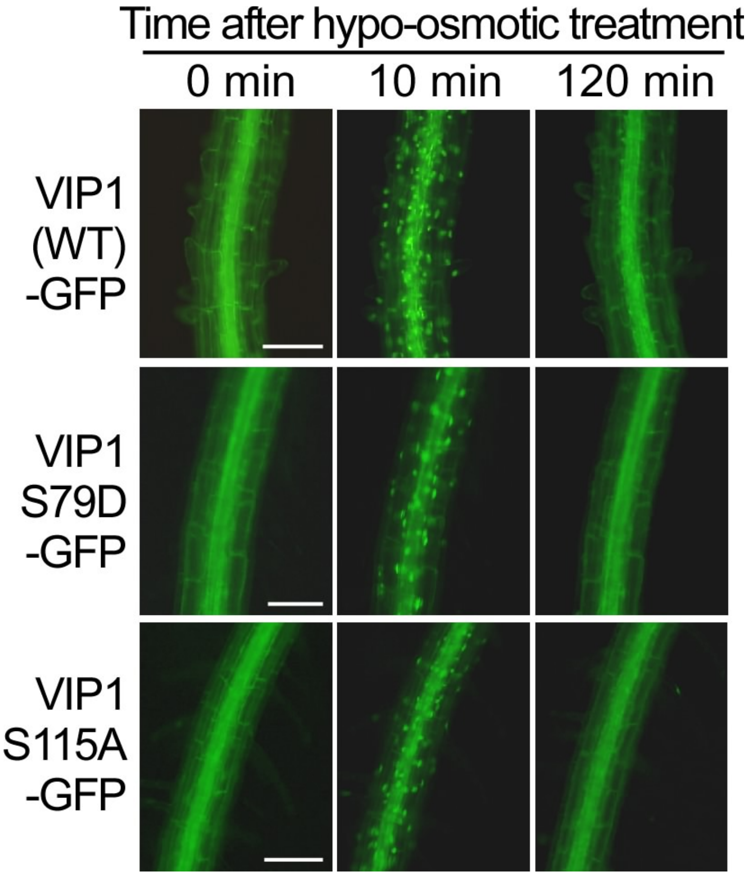

Figure S6. Subcellular localization of VIP1 variants harboring point mutations in the putative phosphorylation sites. A VIP1 variant with either S → D mutation at the amino acid position 79 or S → A mutation at the position 115 (VIP1S79D or VIP1S115A, respectively) was expressed as a GFP-fused protein (VIP1S79D-GFP or VIP1S115A-GFP) in Arabidopsis. Non-mutated VIP1 (VIP1 (WT)-GFP) was used as control. GFP fluorescence in a root was observed 0, 10 and 120 minutes after the root was submerged in 20 mM Tris-HCl, pH 6.8 (hypo-osmotic treatment). More than five individual plants were used for observation in each genotype, and for each construct, representative images in the same root are shown. Scale bars = 100 μm.
