# Supplementary material for: Analysis of Functions of VIP1 and Its Close Homologs in Osmosensory Responses of Arabidopsis thaliana
Source: PLoS One. 2014 Aug 5;9(8):e103930. doi: 10.1371/journal.pone.0103930 (PMC4122391; doi:10.1371/journal.pone.0103930)
Supplement: Figure S7 — Transcriptional activation function of the truncated versions of VIP1 in yeast. (PDF) [file pone.0103930.s007.pdf]

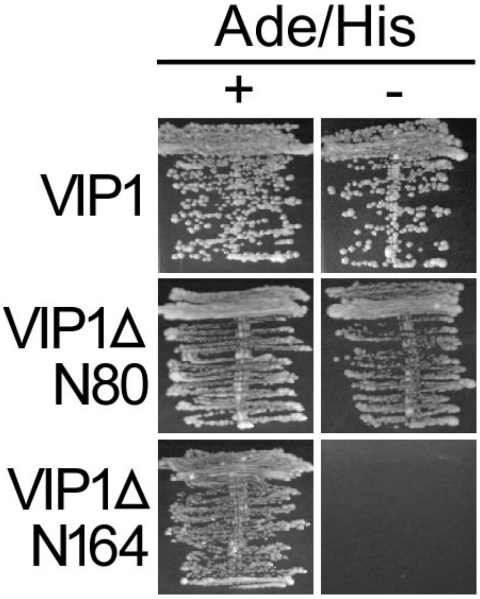

Figure S7. Transcriptional activation functions of the truncated versions of VIP1 in yeast. VIP1 variants lacking the N-terminal 80 amino acids and the N-terminal 164 amino acids (VIP1ΔN80 and VIP1ΔN164, respectively) were expressed as GAL4BD-fused proteins in the yeast strain AH109. Non-truncated VIP1 was used as control (upper panels). Transformed cells were grown on SD media with or without adenine and histidine (Ade/His + or -, respectively). For each construct, growth from three individual colonies was assayed, and a representative result is shown.
