# Supplementary material for: Analysis of Functions of VIP1 and Its Close Homologs in Osmosensory Responses of Arabidopsis thaliana
Source: PLoS One. 2014 Aug 5;9(8):e103930. doi: 10.1371/journal.pone.0103930 (PMC4122391; doi:10.1371/journal.pone.0103930)
Supplement: Figure S8 — An RT-PCR analysis of expression of transcripts for GFP-fused VIP1 variants in Arabidopsis. (PDF) [file pone.0103930.s008.pdf]

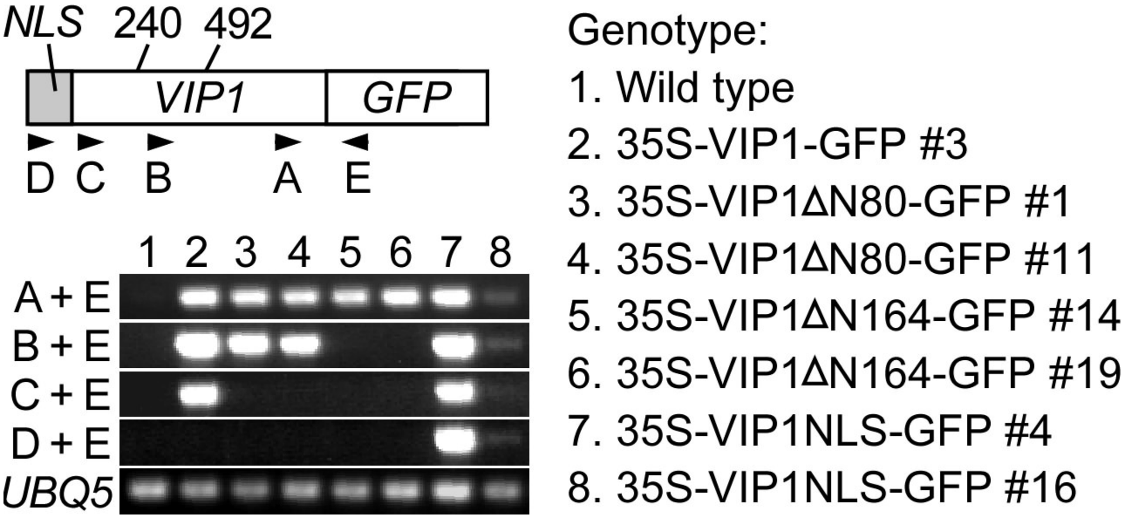

Figure S8. An RT-PCR analysis of expression of transcripts for GFP-fused VIP1 variants in Arabidopsis. Transgenic plants expressing GFP-fused VIP1 variants (35S-VIP1ΔN80-GFP #1, for example) were grown for 10 days, and sampled for RNA extraction and cDNA synthesis. The primers A-E (see the top left panel for their annealing sites in the transgene) were used in the combinations indicated in the bottom left panel. The expression of *UBQ5* is shown as control. The lane numbers correspond to the genotype numbers. Experiments were performed three times and a representative result is shown.
