# Supplementary material for: Analysis of Functions of VIP1 and Its Close Homologs in Osmosensory Responses of Arabidopsis thaliana
Source: PLoS One. 2014 Aug 5;9(8):e103930. doi: 10.1371/journal.pone.0103930 (PMC4122391; doi:10.1371/journal.pone.0103930)
Supplement: Figure S9 — Expression of VIP1 variants do not affect ABA-induced inhibition of seed germination. (PDF) [file pone.0103930.s009.pdf]

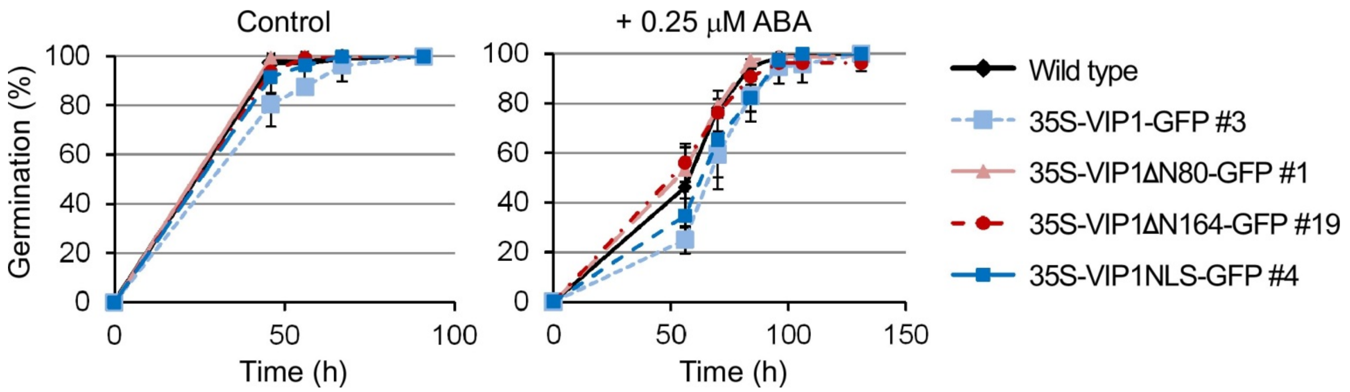

Figure S9. Expression of VIP1 variants does not affect ABA-induced inhibition of seed germination. Seeds were sown on 0.5× MS medium containing 0 (Control) or 0.25 μM ABA, incubated 48 hours at 4 ºC for stratification, and further incubated at 22 ºC. Germinated seeds were counted at the time points indicated after seeds were transferred to the 22 ºC. The experiment was repeated three times, and data were averaged (n = 40 for each experiment). The error bars indicate SD.
