# Supplementary material for: Analysis of Functions of VIP1 and Its Close Homologs in Osmosensory Responses of Arabidopsis thaliana
Source: PLoS One. 2014 Aug 5;9(8):e103930. doi: 10.1371/journal.pone.0103930 (PMC4122391; doi:10.1371/journal.pone.0103930)
Supplement: Table S1 — cDNA clones obtained from RIKEN. (PDF) [file pone.0103930.s010.pdf]

Table S1. cDNA clones obtained from RIKEN

Gene name	TAIR AGI code	RIKEN cDNA clone name
UBQ5	AT3G62250	RAFL06-77-O18
VIP1	AT1G43700	RAFL06-83-I17
AtbZIP52	AT1G06850	RAFL07-12-B18
PosF21	AT2G31370	RAFL09-48-H24
AtbZIP69	AT1G06070	RAFL19-85-B03
AtbZIP29	AT4G38900	RAFL07-09-H03
AtbZIP30	AT2G21230	RAFL07-13-D16
AtbZIP18	AT2G40620	RAFL09-74-P11
