# Supplementary material for: Analysis of Functions of VIP1 and Its Close Homologs in Osmosensory Responses of Arabidopsis thaliana
Source: PLoS One. 2014 Aug 5;9(8):e103930. doi: 10.1371/journal.pone.0103930 (PMC4122391; doi:10.1371/journal.pone.0103930)
Supplement: Table S2 — Primers used for qRT-PCR analyses of expression of Arabidopsis group I bZIP protein genes. (PDF) [file pone.0103930.s011.pdf]

Table S2. Primers used for qRT-PCR analyses of expression of Arabidopsis group I bZIP protein genes

Gene	Name	Sequence (5' > 3')
AtbZIP18	Fw	GCACAAACCCTCCCACCGCT
	Rv	CAAGCCCCTGTAACCGGCCA
AtbZIP52	Fw	GGCCGAATGCAAGGGCTGGA
	Rv	CGTCCTTTCTTGCACAATGGCACC
VIP1	Fw	AGCGCTGCGGGATGAACTGA
	Rv	GGAGCGATGGCTGCCCGTTT
AtbZIP69	Fw	AGGCCACATCTCTCTCAGCCCA
	Rv	TGGACCTTGCCCCGTCAATACCT
PosF21	Fw	GGGCAAACAGGCAGTCCGCA
	Rv	ACGCTGATGGAGCAACTTGGC
AtbZIP29	Fw	CCGTCAATCCGCAGCACGGT
	Rv	ACGCGCTTGTTGCTCCATTGC
AtbZIP30	Fw	GCGAACCGCGTATCTGCTGC
	Rv	TTCGCTGCTGCTGCTCCCAC
UNE4	Fw	CGAGAGTCCGCAGCACATTT
	Rv	AGACATCCCGAAGTTGTGCC
[*]*AtbZIP31*	Fw	AAGAGTCCGCGGCACGTTCA
	Rv	ACGAAGTTCGGCGTGTTGCTCC
	mod1	GTTTTATCATTCTCCAAAAGCTTGATCTTTTC
	mod2	GCTTTTGGAGAATGATAAAACAATGAGGATG
[*]*AtbZIP33*	Fw	GGAGCAACAGGCCCAACTTCGT
	Rv	TCTGTCAAACTCGCGACGGTACT
	mod1	TTTCAGTCAAACCATCACGAAGTTGGGCCTG
	mod2	TCGTGATGCTTTGACTGAAAAATTGCACGT
AtbZIP71	Fw	TGGGAGGTGCAAGCAATGCAA
	Rv	CGCCTTCCCCCACAAAGTTGCT
AtbZIP74	Fw	GCGGCGAGGAAGGCGGATTT
	Rv	ACGTGCAGCTGAGGCTCGGT
UBQ5	Fw	GACGCTTCATCTCGTCC
	Rv	CCACAGGTTGCGTTAG

[*]cDNA fragments for these genes could not be obtained directly by RT-PCR. To generate the fragments corresponding to their cDNA regions, partial fragments of their genomic regions were used first obtained by genomic PCR using primer pairs Fw and Rv, and these PCR products were then used as templates for PCR using either the primer pair Fw and mod1 or the primer pair Rv and mod2. The PCR products obtained with these primer pairs were mixed and used as template for PCR using the primer pair Fw and Rv. The resultant PCR products (corresponding to the partial fragments of cDNAs of *AtbZIP31* and *AtbZIP33*) were purified after agarose gel electrophoresis, and used to make standard curves for qRT-PCR.
