# Supplementary material for: Analysis of Functions of VIP1 and Its Close Homologs in Osmosensory Responses of Arabidopsis thaliana
Source: PLoS One. 2014 Aug 5;9(8):e103930. doi: 10.1371/journal.pone.0103930 (PMC4122391; doi:10.1371/journal.pone.0103930)
Supplement: Table S3 — Primers used for semi-quantitative RT-PCR analyses of transgene expression. (PDF) [file pone.0103930.s012.pdf]

Table S3. Primers used for semi-quantitative RT-PCR analyses of transgene expression

Primer		Sequence (5' > 3')
[*1]VIP1-GFP	Fw	AGCGCTGCGGGATGAACTGA
PosF21-GFP	Fw	GGGCAAACAGGCAGTCCGCA
AtbZIP29-GFP	Fw	CCGTCAATCCGCAGCACGGT
B in Fig. S9	Fw	GTTCGTAGCTTCGCGGTTGATTCCGATTTCTTCGATG
C in Fig. S9	Fw	CCCCACTAGTATGGAAGGAGGAGGAAGAGGACC
D in Fig. S9	Fw	CTCGTCGACATGGATAAAGCGGAATTAATTCCC
[*2]E in Fig. S9	Rv	GTAGGTGGCATCGCCCTCGC

[*1]This is the primer A in Fig. S9

[*2]This primer is *GFP*-specific and was used for all the transgenes.
