# Supplementary material for: Analysis of Functions of VIP1 and Its Close Homologs in Osmosensory Responses of Arabidopsis thaliana
Source: PLoS One. 2014 Aug 5;9(8):e103930. doi: 10.1371/journal.pone.0103930 (PMC4122391; doi:10.1371/journal.pone.0103930)
Supplement: Table S4 — Primer pairs used to generate constructs for Y1H and Y2H assays. (PDF) [file pone.0103930.s013.pdf]

**Table S4.** Primer pairs used to generate constructs for Y1H and Y2H assays

| Gene | | Sequence (5' > 3') (restriction sites are underlined) | Vector and restriction site |
|---|---|---|---|
| *VIP1* | Fw | GCCAGTGAATTCATGGAAGGAGGAGGAAGAGG | pGADT7-Rec<br>*Eco*RI-*Xho*I |
| | Rv | [*1]CCCCGTCGACAGCCTCTCTTGGTGAAATCC | pGBKT7<br>*Eco*RI-*Sal*I |
| *VIP1ΔN80* | Fw | GAGGAATTCATGTCCGTTGATTCGGAAGA | pGBKT7 |
| | Rv | [*1]CCCCGTCGACAGCCTCTCTTGGTGAAATCC | *Eco*RI-*Sal*I |
| *VIP1ΔN164* | Fw | GGTGAATTCTCGATTTTAGCTTCTGTGAGTGG | pGBKT7 |
| | Rv | [*1]CCCCGTCGACAGCCTCTCTTGGTGAAATCC | *Eco*RI-*Sal*I |
| *AtbZIP52* | Fw | CTCCATATGGAGAAATCAGATCCTCC | pGADT7-Rec<br>*Nde*I-*Sac*I |
| | Rv | [*2]GGAAACAGCTATGACCATGATTAC | pGBKT7<br>*Nde*I-*Sal*I |
| *AtbZIP69* | Fw | CTCCATATGGATAAGGAGAAATCTCCTGC | pGADT7-Rec<br>*Nde*I-*Bam*HI |
| | Rv | [*2]GGAAACAGCTATGACCATGATTAC | pGBKT7<br>*Nde*I-*Bam*HI |
| *PosF21* | Fw | CCACCCATGGATAAGGAGAAATCTCCAGC | pGADT7-Rec<br>*Nco*I-*Xba*I |
| | Rv | GGGACTAGTGTTCTCTTTCTGGGCTTGTG | pGBKT7<br>*Nco*I-*Sal*I |
| *AtbZIP29* | Fw | CCTCATATGGGTGATACAGAGAAGTGT | pGADT7-Rec<br>*Nde*I-*Bam*HI |
| | Rv | [*2]GGAAACAGCTATGACCATGATTAC | pGBKT7<br>*Nde*I-*Bam*HI |
| *AtbZIP30* | Fw | CTCCATATGGGTGGTGGTGGTGATAC | pGADT7-Rec<br>*Nde*I-*Bam*HI |
| | Rv | [*2]GGAAACAGCTATGACCATGATTAC | pGBKT7<br>*Nde*I-*Bam*HI |

[*1]These are identical and specific to the 3' end of *VIP1* ORF.

[*2]These are identical and specific to the sequence of the vector for RIKEN cDNA clones. These clones have either a *Bam*HI or a *Sac*I site at the 3'-flanking region of cDNA sequences. The ORF of *AtbZIP52* were amplified by PCR using the RIKEN cDNA clone as template and the listed primers, and inserted into the *Nde*I-*Sac*I site of pGADT7-Rec. This construct was digested by *Nde*I and *Xho*I, and the resultant DNA fragment containing *AtbZIP52* ORF was inserted into the *Nde*I-*Sal*I site of pGBKT7. The ORFs of *AtbZIP69*, *AtbZIP29* and *AtbZIP30* were obtained by PCR using the RIKEN cDNA clones as template and listed primers, digested by *Nde*I and *Bam*HI, and inserted into the *Nde*I-*Bam*HI site of pGADT7-Rec and pGBKT7.
