# Supplementary material for: Analysis of Functions of VIP1 and Its Close Homologs in Osmosensory Responses of Arabidopsis thaliana
Source: PLoS One. 2014 Aug 5;9(8):e103930. doi: 10.1371/journal.pone.0103930 (PMC4122391; doi:10.1371/journal.pone.0103930)
Supplement: Table S5 — Primer pairs used to generate constructs for expression of nYFP-, cYFP- and GFP-fused proteins. (PDF) [file pone.0103930.s014.pdf]

Table S5. Primer pairs used to generate constructs for expression of nYFP-, cYFP- and GFP-fused proteins

Gene		Sequence (5' > 3') (restriction sites are underlined)	[*1]Vector and restriction site
[*3]*VIP1*<br>[*3]*VIP1ΔN20*<br>[*4]*VIP1ΔN80*	Fw	CCCCACTAGTCATATGGAAGGAGGAGGAAGAGGACC	pBS-35S-nYFP-2<br>[*3]pBS-35SMSC-GFP
	Rv	[*2]CCCCGTCGACAGCCTCTCTTGGTGAAATCC	pBI121-35SMCS-GFP<br>*Xba*I-*Sal*I
VIP1ΔN50	Fw	CCTACTAGTCATATGGATCCTTCCGATATCGATTTCTC	pBS-35SMSC-GFP
	Rv	[*2]CCCCGTCGACAGCCTCTCTTGGTGAAATCC	pBI121-35SMCS-GFP<br>*Xba*I-*Sal*I
VIP1ΔN164	Fw	GGTTCTAGAATGTCGATTTTAGCTTCTGTGAGTGG	pBS-35SMSC-GFP
	Rv	[*2]CCCCGTCGACAGCCTCTCTTGGTGAAATCC	pBI121-35SMCS-GFP<br>*Xba*I-*Sal*I
PosF21	Fw	CCCACTAGTATGGATAAGGAGAAATCTCCAGC	pBS-35SMCS-cYFP<br>pBS-35SMCS-GFP
	Rv	GGGACTAGTGTTCTCTTTCTGGGCTTGTG	pBI121-35SMCS-GFP<br>*Spe*I
AtbZIP29	Fw	GAGGTCGACATGGGTGATACAGAGAAGTGT	pBS-35SMCS-cYFP<br>pBS-35SMCS-GFP
	Rv	CCGACTAGTTTCATTTGATTCAGATTTTGTTGCC	pBI121-35SMCS-GFP<br>*Sal*I-*Spe*I
AtbZIP52	Fw	CCCGTCGACATGGAGAAATCAGATCCTCCACC	pBS-35SMCS-cYFP
	Rv	CCCACTAGTATAGGCAGAGCTACTCTCACTAGC	pBS-35SMCS-GFP<br>*Sal*I-*Spe*I
AtbZIP31	Fw	CCCGTCGACATGAATGGATCCGACAATAGC	pBS-35SMCS-GFP
	Rv	GGGACTAGTCATGTTGTCATCCCTAGATTTTGG	*Sal*I-*Spe*I
UNE4	Fw	CCCGTCGACATGAATGGATCCGACAATACC	pBS-35SMCS-GFP
	Rv	GTGACTAGTTCCATTAAAACCAGGGTTTGGT	*Sal*I-*Spe*I
AtbZIP33	Fw	GAGGTCGACATGAATGGATCCGACAATATCA	pBS-35SMCS-GFP
	Rv	GGGACTAGTAATCTGTCCATAAAAACCAAGG	*Sal*I-*Spe*I

[*1]All the restriction sites used are shared among all the vectors listed.

[*2]These are identical.

[*3] The full-length ORF of VIP1 was amplified by PCR using the RIKEN cDNA clone as template and the listed primers, digested by *Spe*I and *Sal*I, and inserted into the *Xba*I-*Sal*I site of pBS-35SMCS-GFP. This construct was digested by *Nde*I, and the resultant larger DNA fragment was self-ligated to remove the 5' ~50 bp of VIP1 ORF. The resultant plasmid is pBS-35SMCS-GFP containing *VIP1ΔN20*.

[*4]To generate the construct for expressing VIP1ΔN80, the PCR products obtained with VIP1 Fw and Rv primers were digested by *Sal*I and *Xba*I (a *Xba*I site is present at the position 178 from the start codon of the ORF of *VIP1*). The resultant fragment was inserted into the *Spe*I-*Sal*I site of the relevant vectors.
