# Supplementary material for: Analysis of Functions of VIP1 and Its Close Homologs in Osmosensory Responses of Arabidopsis thaliana
Source: PLoS One. 2014 Aug 5;9(8):e103930. doi: 10.1371/journal.pone.0103930 (PMC4122391; doi:10.1371/journal.pone.0103930)
Supplement: Table S6 — Primers used to generate the point-mutated versions and the NLS-attached version of VIP1. (PDF) [file pone.0103930.s015.pdf]

Table S6. Primers used to generate the point-mutated versions and the NLS-attached version of VIP1

Gene	Primer name	Sequence (5' > 3') (restriction sites are underlined)	Vector and restriction site
[*3]*VIP1S79D*	VIP1Fw	[*1]CCCCACTAGTATGGAAGGAGGAGGAAGAGGACC	
VIP1S115A	VIP1Rv	[*2]CCCCGTCGACAGCCTCTCTTGGTGAAATCC	
	S79D-1	CGGACATGGGATCAGCTTGCGGTTGTTGTTGTGATTG	pBI121-35SMCS-GFP *Xba*I-*Sal*I
	S79D-2	CCGCAAGCTGATCCCATGTCCGTTGATTCGGAAG	
	S115A-1	CGGAATCAACCGCGAAGCTACGAACATGGCGACCG	
	S115A-2	GTTCGTAGCTTCGCGGTTGATTCCGATTTCTTCGATG	
[*4]*VIP1NLS*	VIP1Fw	[*1]CCCCACTAGTATGGAAGGAGGAGGAAGAGGACC	pBS-35SMSC-GFP
	VIP1Rv	[*2]CCCCGTCGACAGCCTCTCTTGGTGAAATCC	
	AD5'Fw	CTCGTCGACATGGATAAAGCGGAATTAATTCCC	pBI121-35SMCS-GFP *Sal*I
	VIP1NLS Rv	GTCCTCTTCCTCCTCCTTCCATGGCGGTACCCAATTCGACCT	

[*1]These are identical.

[*2]These are identical.

[*3]To generate the ORF of *VIP1S79D*, the RIKEN cDNA clone for *VIP1* was used as template for PCR using either the primer pair VIP1Fw and S79D-1 or the primer pair VIP1Rv and S79D-2. The PCR products obtained with these primer pairs were mixed and used as template for PCR using the primer pair VIP1Fw and VIP1Rv. The resultant PCR products correspond to the ORF of *VIP1S79D*. The ORF of VIP1S115A was generated in the same way except that the primers S115A-1 and S115A-2 were used instead of the primers S79D-1 and S79D-2, respectively.

[*4]To generate the ORF of VIP1NLS, pGADT7-Rec containing *VIP1* (described above) was used as template for PCR using either the primer pair AD5'Fw and VIP1NLS Rv or the primer pair VIP1Fw and VIP1Rv. The PCR products obtained with these primer pairs were mixed and used as template for PCR using the primer pair AD5'Fw and VIP1Rv. The resultant PCR products correspond to the ORF of *VIP1NLS*. The ORF of VIP1S115A was generated in the same way except that the primers S115A-1 and S115A-2 were used instead of the primers S79D-1 and S79D-2, respectively.
